# A Small Molecule That Inhibits the Quorum Sensing Receptor AgrC in *Staphylococcus aureus*

**DOI:** 10.1021/jacs.5c21051

**Published:** 2026-02-23

**Authors:** Thomas J. Polaske, Troy D. Vulpis, Alexandra E. Nelson, Ke Zhao, Helen E. Blackwell

**Affiliations:** Department of Chemistry, 5228 University of Wisconsin−Madison, 1101 University Ave., Madison, Wisconsin 53706, United States

## Abstract

We report the synthesis and characterization of a small molecule, CP-20, capable of inhibiting the key quorum sensing receptor AgrC in *Staphylococcus aureus*. Structural alterations enabled the discovery of a CP-20 derivative that is capable of complete quorum sensing inhibition with mid-nanomolar potency. *In vitro* biochemical investigations support CP-20 binding AgrC and competitively inhibiting its activity. CP-20 represents a versatile and synthetically tractable small molecule probe for investigating Staphylococcal virulence via quorum sensing modulation.

## Graphical abstract

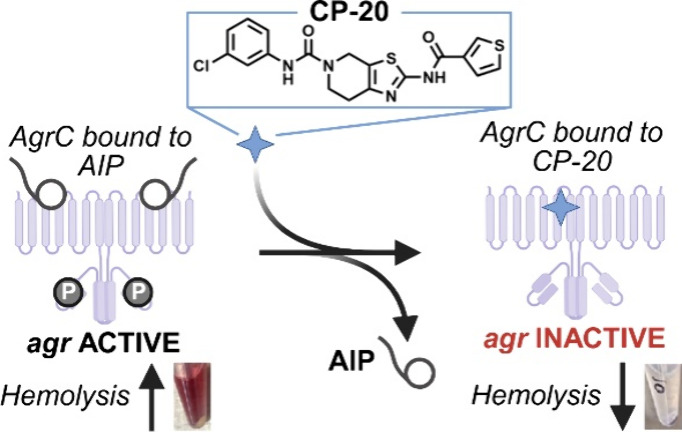

*Staphylococcus aureus* is a formidable bacterial pathogen known for its ability to cause life-threatening antibiotic-resistant infections.^1,2^ Using the accessory gene regulator (*agr*) quorum sensing (QS) system (Figure 1A), *S. aureus* can monitor its population density and trigger acute infection at threshold cell numbers.^3,4^ This chemical sensing system is based on the production of an autoinducing peptide (AIP) signal and its recognition via a sensor histidine kinase, AgrC. Several *in vivo* studies using both *agr*-null mutants and chemical *agr* inhibitors have illustrated the importance of a functional *agr* system in establishing infection.^5–8^ Accordingly, the *agr* QS circuit has attracted significant attention over the past two decades as a target for antivirulence strategies.^9–16^ Many of these approaches have been based on macrocyclic peptides that closely mimic the AIP signal and are believed to act on AgrC (Figure 1B).^13,14^ Although these peptides have proven useful probes, there are limits with regard to their hydrolytic and metabolic stability, synthetic accessibility, and ability to traverse host cell membranes to target intracellular *S. aureus*.^18–20^ Small molecule *agr* inhibitors have been reported, but their potencies are dramatically weaker than peptide-based modulators (∼1000-fold), and many have unclear mechanisms of action and associated toxicity.^21–24^ Potent, physically robust, and synthetically tractable small molecule *agr* inhibitors with defined cellular targets would represent a significant advance for the study of QS. We describe the characterization of such a compound herein.

**Figure 1:**
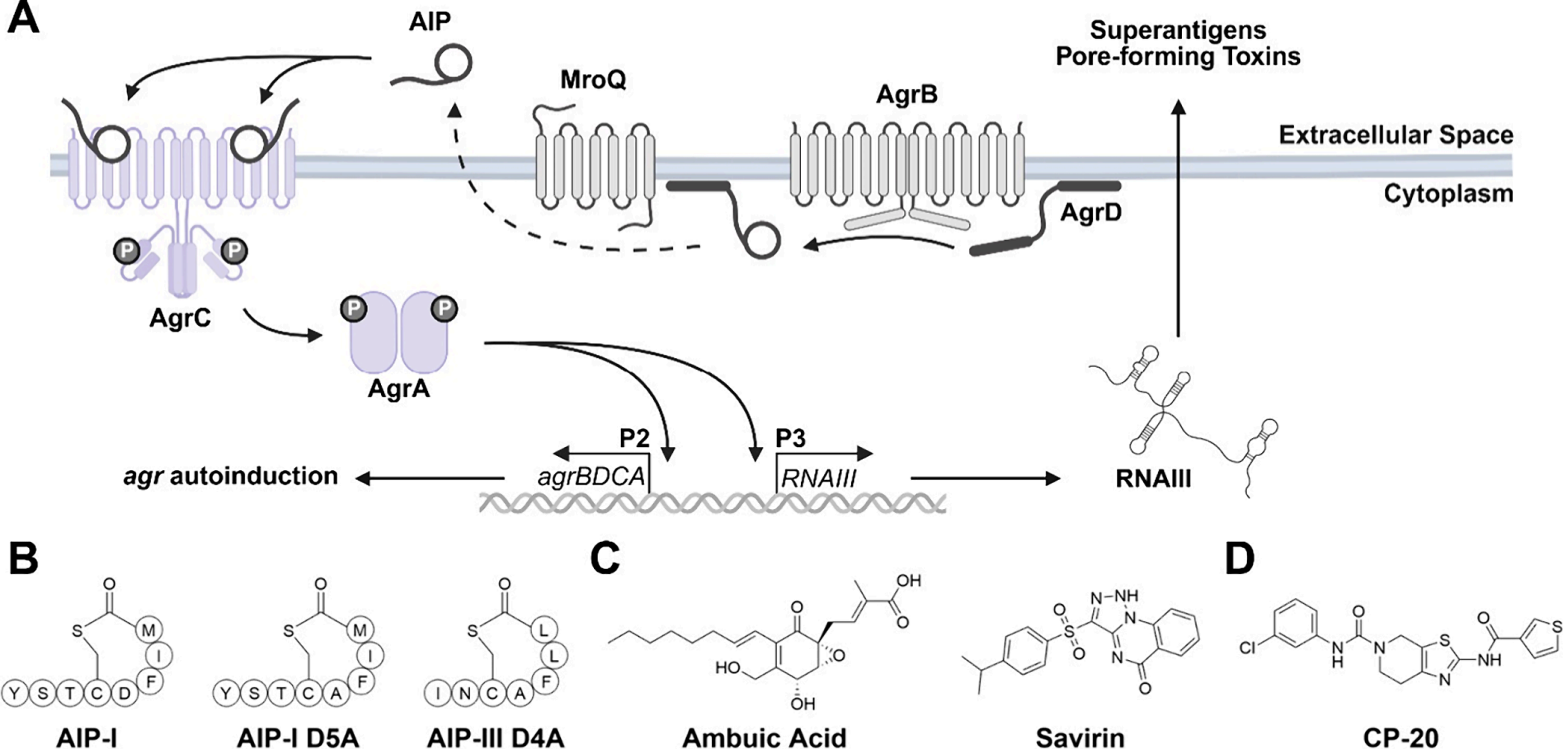
(A) Schematic of the accessory gene regulator (*agr*) quorum sensing system in *S. aureus*. Structures of (B) the *S. aureus* AIP-I signal and the peptidic *agr* inhibitors, AIP-I D5A and AIP-III D4A (one letter codes used for amino acids), (C) representative small molecule *agr* inhibitors, ambuic acid and savirin, and (D) CP-20.

The *agr* system is found in the Staphylococci and many closely related species, but best understood in *S. aureus*.^25^ This signaling pathway is composed of four proteinaceous components, each of which represents a target for *agr* modulation (Figure 1A).^3^ The AIP signal (Figure 1B) is biosynthesized via the proteolytic processing of the proto-peptide, AgrD, by AgrB and (in most *S. aureus* strains) MroQ, and exits the cell.^26–30^ Upon reaching quorate population, the local extracellular AIP concentration reaches sufficient levels for productive binding to the sensor domain of the transmembrane receptor histidine kinase, AgrC, triggering its autophosphorylation and intracellular phospho-transfer to its partner response regulator, AgrA.^31^ Phosphorylated AgrA then upregulates production of numerous secreted toxins, exoenzymes, hemolysins, and other virulence factors, along with the *agr* machinery, thereby completing the QS autoinduction loop.^32–34^ An interesting feature of the *agr* system is the subspeciation of *S. aureus* into four *agr* subtypes (groups-I–IV) in which four divergent AIP signals have coevolved to recognize their cognate AgrC receptors.^34,35^

Of the known small molecules that inhibit the *S. aureus* *agr* system and examined mechanistically on any level, almost all target AgrA (e.g., savirin^21^), with ambuic acid^22^ the lone probe reported to block AIP biosynthesis (Figure 1C). Recently, we reported the discovery of a small molecule, CP-20 (Figure 1D), capable of blocking virulence factor production in *S. aureus*.^36^ Follow-up experiments using a group-I *S. aureus* transcriptional reporter that produces yellow fluorescent protein under the control of the *agr* P3 promoter (Figure 1A) demonstrated that CP-20 inhibits *agr*. This activity was recapitulated in a secondary assay showing CP-20 completely blocked hemolysin production in a group-I MRSA strain (USA300 LAC) (Figure S1). In view of CP-20’s strong activity and novel structure, we sought to further delineate its mechanism of *agr* inhibition.

We first explored the activity of CP-20 in *S. aureus* groups-II–IV and conducted cell-based transcriptional reporter assays analogous to those described for group-I (Figure S2).^36^ Savirin was used as a positive control, as it represents the most potent small molecule *agr* inhibitor available. CP-20 was comparable to or more potent than savirin across all four groups, with single-digit micromolar half-maximal inhibitory concentrations (IC_50_ values; Table 1). Although CP-20 displayed similar efficacies in *agr* groups-I, -III, and -IV (>90% max. inhibition), it was notably less active in group-II (49% max. inhibition). This discrepancy suggested that CP-20 could target a divergent portion of the *agr* system, such as AgrC, as the AgrC sensor domain (∼30–55% conserved across *agr* subtypes, Figure S3) is known to tightly discriminate between different native AIP signals.^3^

**Table 1:** *S. aureus* and *S. epidermidis* *agr* Fluorescence Reporter Assay Data for CP-20 and Savirin

	CP-20		Savirin	
Species/*agr* group	IC_50_ [μM] *(95% CI)*	Max. Inhibition [%]	IC_50_ [μM] *(95% CI)*	Max. Inhibition [%]
*S.a*.-I	0.93 *(0.79–1.1)*	98	9.2 *(8.7–9.6)*	98
*S.a*.-II	5.1 *(3.1–9.7)*	49	7.2 *(5.7–9.1)*	99
*S.a*.-III	1.0 *(0.75–1.4)*	100	15 *(n.c.)*	99
*S.a*.-IV	3.5 *(2.8–4.3)*	91	7.4 *(6.6–8.1)*	95
*S.e*.-I	11 (*n.c*.)	43	96^a^ (*n.c*.)	90^a^
*S.e*.-II	2.3^b^ (*n.c*.)	450^b^	2.9^a^ (*2.6–3.3*)	92^a^

a Significant growth defects observed at high concentrations of savirin.

b Values report EC_50_ [μM] (*95% CI*) and maximal activation [%]. n.c. = not calculated, an upper or lower limit could not be calculated to 95% confidence.

Next, we examined the ability of CP-20 to modulate *agr* in a closely related pathogen, *Staphylococcus epidermidis*. Like *S. aureus*, *S. epidermidis* has multiple *agr* subtypes, of which we tested groups-I and -II, again using cell-based transcriptional reporter assays (Table 1). Interestingly, CP-20 showed dissimilar activities in the two groups, exhibiting moderate inhibition in *S. epidermidis* group-I yet acting as a superactivator in group-II (Figure S2B,C). Super-agonism has been previously demonstrated in these two *S. epidermidis* reporters with both native AIPs and structurally related AIP analogs,^37^ compound classes that act via the AgrC sensor domain. This activity profile for CP-20 also hinted that it could potentially target the AgrC sensor domain in both *S. epidermidis* and *S. aureus* (∼30–40% conserved across species, Figure S3).^37,38^

We next investigated the effects of structural changes to the CP-20 scaffold on its *agr* inhibitory activity. We developed a straightforward four-step synthetic route toward CP-20 (Figure 2A) that assembles the core via a thiazole condensation, followed by amide and urea formations, providing product in ∼ 60% overall yield.^39–42^ Inspired by the *meta*-chloro aryl substituent in CP-20, we substituted Cl for F, Br, and I to yield CP-20-F, -Br, and -I, respectively. Evaluation of these derivatives in the *agr* reporter assay revealed inhibitory activity increased with halogen size, with CP-20-I 5-fold more potent than CP-20 in *S. aureus agr* group-I (IC_50_ = 180 nM), making it the most potent small molecule *S. aureus agr* inhibitor reported to date (Figure 2B, Table S2). In turn, introduction of the fluoride in CP-20-F caused a nearly 5-fold reduction in potency. These results indicate that the CP-20 scaffold is tunable for *agr* inhibition.

**Figure 2:**
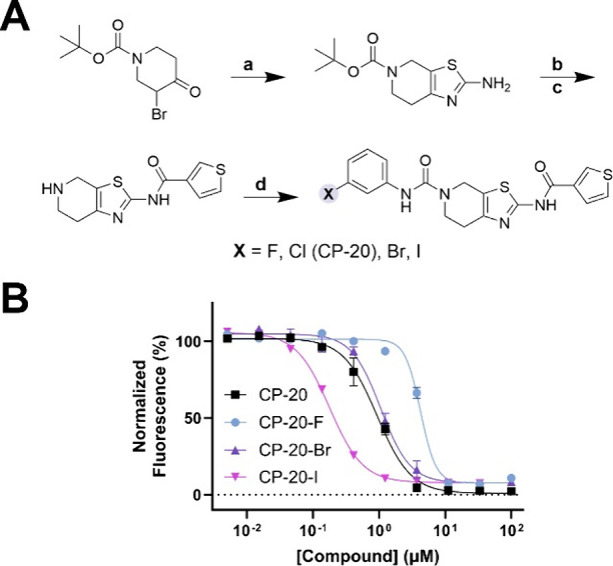
(A) Synthesis of CP-20 and halogen derivatives. Reagents: (a) thiourea, IPA, Δ, 98%; (b) thiophene-3-carboxylic acid, EDC-HCl, DMAP, TEA, DCM, rt, 75%; (c) TFA, DCM, rt; (d) *N*-Boc-3-haloaniline, DABAL-Me_3_, toluene, Δ, 82%. (B) Fluorescent transcriptional reporter assay (*S. aureus* *agr*-I) for CP-20 derivatives. Data represent mean ± s.e.m. for three biological replicates.

To further narrow the pathways by which CP-20 can block *agr* activity, we subjected it to an array of cell-based and *in vitro* assays. First, we examined CP-20’s activity in a group-I *S. aureus* reporter that isolates the AgrC/A signal transduction system in an *agr*-null background and measures activity via β-lactamase at the P3 promoter.^15^ We used the native *S. aureus* AIP-I to activate the system and the competitive peptide-based AgrC inhibitor, AIP-III D4A (Figure 1B), and the small molecule AgrA inhibitor, savirin, as positive controls.^13,21^ Ambuic acid served as a negative control, as it should not affect AgrC/A signal transduction.^43^ We observed CP-20 to completely inhibit P3 transcription in this assay (IC_50_ = 16 μM), strongly suggesting it targets either AgrC or AgrA (Figure 3A). In addition, CP-20-I was approximately 5-fold more potent than CP-20 in this assay (IC_50_ = 3.5 μM, Figure S4), aligning with its activity profile in the group-I reporter containing a complete *agr* system.

**Figure 3:**
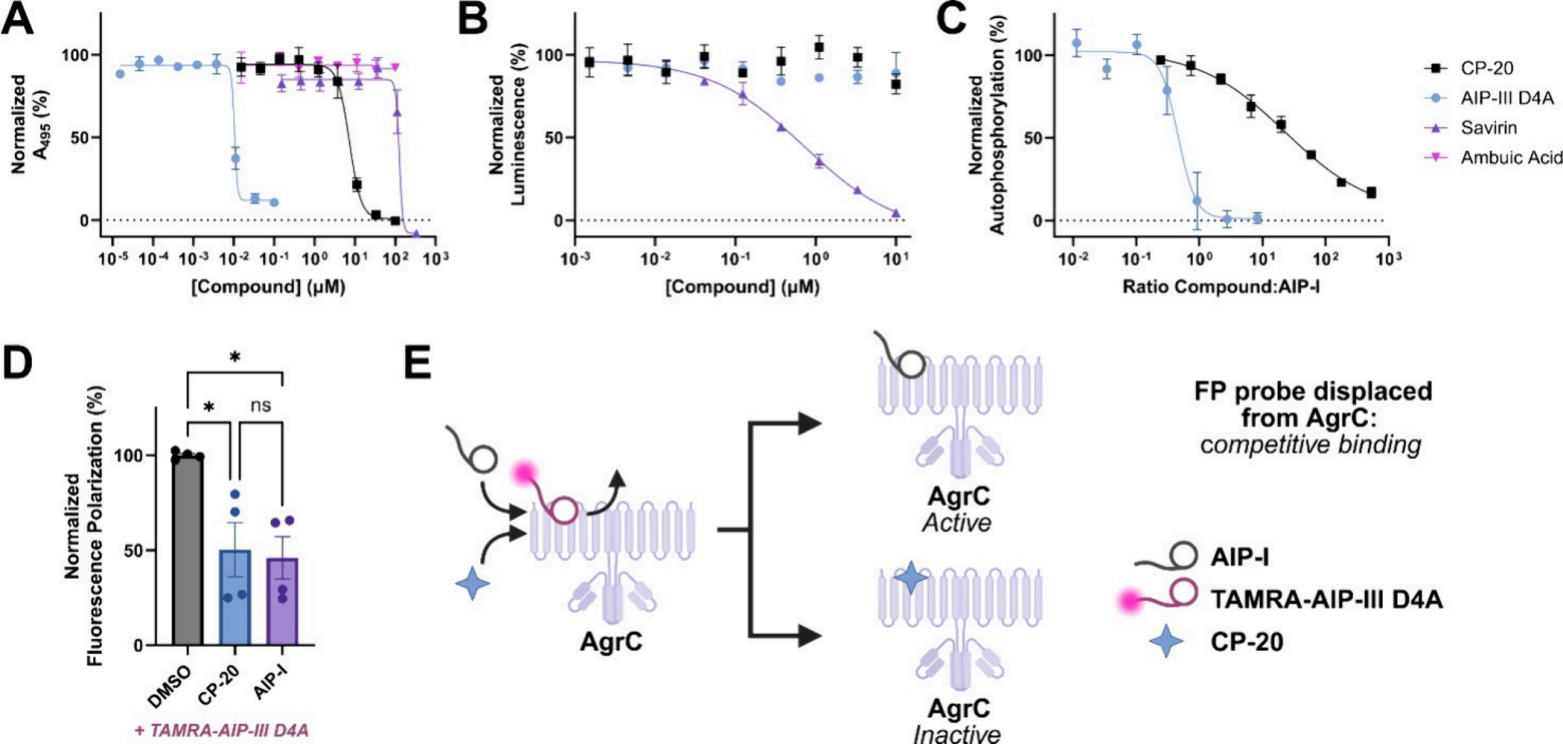
CP-20 *agr* target identification. (A) β-Lactamase reporter data from a *S. aureus* strain that measures AgrC/A activity in the presence of compound. AgrC activated by added AIP-I (100 nM) and β-lactamase activity measured via cleavage of the substrate, nitrocefin (λ_max_ of approximately 495 nm). (B) Luminescent reporter data from a *S. aureus* strain that measures AgrA activity in the presence of compound. (C) *In vitro* AgrC autophosphorylation data in competition with AIP-I (3.5 μM for CP-20 and 20 μM for AIP-III D4A). (D) *In vitro* fluorescence polarization data for the displacement of TAMRA-AIP-III D4A (375 nM) from AgrC by AIP-I and CP-20 (both at 10 μM) or DMSO control. (E) Schematic of proposed mechanism of competitive AgrC inhibition by CP-20. Data represent the mean ± s.e.m. for at least three biological replicates. *p < 0.05 by an ordinary one-way ANOVA with Turkey’s multiple comparisons test.

To differentiate between these two potential targets, we used a *S. aureus* AgrA reporter construct in which the *agr* operon has been replaced by a luciferase reporter, and AgrA (group-I) is expressed from a plasmid.^21^ AgrA:DNA binding thus produces measurable luminescence output. As a key positive control, savirin was found to block AgrA activity in this assay, as expected (Figure 3B).^21^ However, we observed CP-20 treatment had no significant effect on luminescence (Figure 3B). This result suggested that CP-20 does not inhibit *agr* by directly preventing AgrA from binding to DNA.

Narrowing our focus to AgrC, we utilized an *in vitro* method developed by Muir and co-workers that uses radiolabeled ATP to monitor AgrC activation (i.e., autophosphorylation) in the presence of AIP.^31^ The assay uses AgrC nanodiscs, in which a dimer of purified AgrC is isolated inside a nanometer-scale phospholipid disc held together by a membrane scaffold protein, thereby stabilizing AgrC in its functional form and allowing for direct measurement of autophosphorylation *in vitro* (Figure S5). Using the AgrC inhibitor AIP-III D4A and DMSO as positive and negative controls, respectively, we found that CP-20 caused a dose-dependent decrease in autophosphorylation when coincubated with AIP-I (Figure 3C). This result supports the ability of CP-20 to directly inhibit AgrC autophosphorylation in the absence of other *agr* machinery. To the best of our knowledge, CP-20 is the first synthetic small molecule reported to inhibit AgrC.

To probe the mechanism by which CP-20 inhibits AgrC, we next conducted *in vitro* fluorescence polarization (FP) assays using AgrC nanodiscs and an N-terminal tetramethylrhodamine-labeled AIP-III D4A derivative (TAMRA-AIP-III D4A). We validated that TAMRA-AIP-III D4A inhibits group-I *agr* in fluorescent transcriptional reporter assays (IC_50_ = 28.8 nM) and found that its potency was reduced upon exogenous addition of the native signal, AIP-I, in a concentration-dependent manner (Figure S6A). This result suggests that TAMRA-AIP-III D4A competitively inhibits AgrC in cells and supported its use as an FP probe. In the absence of competing ligand, the interaction between AgrC and TAMRA-AIP-III D4A yielded a high FP signal; treatment with AIP-I led to a 62% reduction in polarization (Figure 3D-E), supporting the displacement of TAMRA-AIP-III D4A from AgrC by AIP-I *in vitro*. Treatment with CP-20 similarly reduced polarization by 52%, suggesting that it also displaces TAMRA-AIP-III D4A upon binding to AgrC (Figure 3D-E). Together, these data support the premise that CP-20 inhibits AgrC via a competitive binding mechanism. This competitive mechanism is further supported by cell-based *agr* reporter assays in which CP-20’s IC_50_ increases upon treatment with increasing concentrations of synthetic AIP-I, while its efficacy is unchanged (Figure S6B).

Additional experiments were performed to begin to unravel the molecular mechanisms by which CP-20 inhibits AgrC activity. CP-20’s varied inhibitory activity across *S. aureus* subtypes and ability to activate *agr* in *S. epidermidis* group-II imply that it likely targets the divergent AgrC sensor domain. Hill slope analysis of the *in vitro* autophosphorylation data (Figure 3C) suggests that CP-20 (Hill coefficient = −0.63 ± 0.41) functions in a less cooperative manner than AIP-III D4A (Hill coefficient = −3.1 ± 1.7), perhaps representing a distinct mode of inhibition from traditional peptide-based AgrC inhibitors. We performed computational experiments using DiffDock to examine putative binding sites for CP-20 on an Alphafold2-generated model of *S. aureus* AgrC (group-I).^44,45^ These experiments predicted that CP-20 binds to a membrane-exposed portion of the AgrC sensor domain rather than the extracellular-facing ligand binding domain at which AIP-I and AIP-III D4A are predicted to interact (Figure S7). Such a model suggests that CP-20 may access binding pockets that are inaccessible to peptide-based inhibitors. Future work using reactive CP-20 derivatives capable of labeling specific AgrC residues at which they interact could illuminate such pathways and are ongoing.

To close, CP-20’s small molecule structure and synthetic tractability make it an exciting addition to the historically peptide-dominated chemical toolbox of AgrC modulators. Simple structural modifications revealed CP-20-I—with 5-fold higher potency than CP-20—demonstrating that this scaffold likely can be further tuned to yield IC_50_s that approach those of lead peptidic *agr* inhibitors (with low- and sub-nanomolar activities). We anticipate the CP-20 scaffold is more host cell penetrant and chemically stable than peptidic *agr* inhibitors, while still maintaining similar bioactivity. Studies to probe such properties will be reported in due course.

## Supplementary Material



## Notes

Experimental details, compound characterization, and additional biological data (PDF)

## †

PPD, 3230 Deming Way, Middleton, WI 53562, United States

## §

Alexion Pharmaceuticals, 100 College St., New Haven, CT 06510, United States
