# Supplementary material for: A Small Molecule That Inhibits the Quorum Sensing Receptor AgrC in Staphylococcus aureus
Source: J Am Chem Soc. 2026 Feb 23;148(9):9199–204. doi: 10.1021/jacs.5c21051 (PMC12983307; doi:10.1021/jacs.5c21051)
Supplement: Supplementary file 1 [file ja5c21051_si_001.pdf]

SUPPORTING INFORMATION

A Small Molecule that Inhibits the Quorum Sensing Receptor AgrC in *Staphylococcus aureus*

Thomas J. Polaske,[‡,†] Troy D. Vulpis,[1,‡] Alexandra E. Nelson, Ke Zhao,[§] and Helen E. Blackwell*

Department of Chemistry, University of Wisconsin–Madison, 1101 University Ave., Madison, WI 53706, USA

**Correspondence:* blackwell@chem.wisc.edu

CONTENTS.

- Chemistry materials and methods
- Synthesis of CP-20 derivatives and compound characterization data
- Synthesis of peptide probes and compound characterization data
- General biological reagents and methods
- **Table S1.** Bacterial strains and plasmids used in this study
- Bacterial reporter assay methods
- Protein production and *in vitro* assay methods
- Hemolysis assay protocol
- **Figure S1.** Rabbit red blood cell (RBC) hemolysis assay data
- Additional text for **Figure S1**
- **Figure S2.** Fluorescent reporter assays for CP-20 and savirin
- **Figure S3.** AgrC multiple sequence alignment analysis
- **Table S2.** *S. aureus* fluorescent reporter assays for CP-20 halogen derivatives
- **Figure S4.** Cell-based AgrC/AgrA activity assays for CP-20 and CP-20-I
- **Figure S5.** Characterization data for purified recombinant proteins and AgrC-I nanodiscs
- **Figure S6.** Cell-based competitive *agr* inhibition assays with TAMRA-AIP-III D4A and CP-20
- Computational methods
- **Figure S7.** Views of computational models of ligand:AgrC-I binding
- **Figure S8.** Uncropped gel images from protein and nanodisc purification
- References

Present Addresses
[†]T.J.P. — *Present Address: PPD, 3230 Deming Way, Middleton, WI 53562, USA*
[§]K.Z. — *Present Address: Alexion Pharmaceuticals, 100 College St., New Haven, CT 06510, USA*

Author Contributions
[‡]T.J.P. and T.D.V. contributed equally.

Chemistry materials and methods.

Reagents. Solvents used for synthesis and purification—dichloromethane (DCM, HPLC grade), diethyl ether (ACS reagent), ethyl acetate (EtOAc, HPLC grade), hexanes (ACS reagent), isopropanol (IPA, ACS reagent), tetrahydrofuran (THF, anhydrous-inhibitor free), toluene (ACS reagent), and dimethylformamide (DMF, ACS reagent)—were purchased from Sigma-Aldrich and used without any further purification. Sodium chloride, sodium hydroxide, hydrochloric acid, magnesium sulfate, imidazole, bis(trimethylaluminum)-1,4-diazabicyclo[2.2.2]octane adduct (DABAL-Me_3), 3-fluoroaniline, 3-chloroaniline, 3-bromoaniline, 3-iodoaniline, 4-(dimethylamino)pyridine (DMAP), di-*tert*-butyl dicarbonate, silica supported perchloric acid, trifluoroacetic acid (TFA), piperidine, triethylamine (TEA), and diisopropylethylamine (DIPEA) were also purchased from Sigma-Aldrich. *Tert*-butyl 3-bromo-4-oxopiperidine-1-carboxylate was purchased from Ambeed, Inc. Thiourea and 4-nitrophenyl chloroformate were purchased from Acros Organics. Thiophene 3-carboxylic acid was purchased from Santa Cruz Biotechnology. Ethanol (EtOH) was purchased from Decon Labs. All Fmoc- and Boc-protected amino acids, 3-(3-dimethylaminopropyl)-1-ethyl-carbodiimide hydrochloride (EDC-HCl), *O*-(7-Azabenzotriazol-1-yl)-*N*,*N*,*N'*,*N'*-tetramethyluronium hexafluorophosphate (HATU), and 5(6)-carboxytetramethylrhodamine (TAMRA) were purchased from Chem Impex. Dawson Dbz AM resin was purchased from Novabiochem. Deuterated solvents for nuclear magnetic resonance (NMR) experiments were purchased from Sigma-Aldrich. Savirin was purchased from AK Scientific. Ambuic acid was purchased from Neta Scientific.

TLC visualization was performed using UV light (254 nm) and iodine. All synthesized compounds were determined to have >95% purity via NMR (for CP-20 and its derivatives) or high-performance liquid chromatography (HPLC) with detection by UV absorbance at 220 nm (for peptides).

Instrumentation. NMR spectra were collected using deuterated solvents at 400 MHz on a Bruker-Avance spectrometer equipped with BFO probe and at 500 MHz on a Bruker-Avance spectrometer equipped with a DCH cryoprobe. Chemical shifts are reported in ppm using residual solvent peaks or tetramethylsilane (TMS) as reference. Coupling constants are given in Hz. Electrospray ionization-exact mass measurement (ESI-EMM) MS data were collected on a Thermo Q-Exactive PLUS ESI-Q-Orbitrap mass spectrometer or a Thermo Q-Exactive FOCUS ESI-Q-Orbitrap mass spectrometer. Reverse-phase HPLC was performed on a Shimadzu system equipped with a SLC-10Avp controller, a LC-20AT prominence pump, a DGU-20A5R degassing unit, a SIL-10AF auto sampler, an FRC-10A fraction collector, a CTO-20A prominence column oven, and a SPC-M20A prominence UV/Vis diode array detector.

<u>Synthesis of CP-20 derivatives and compound characterization data.</u>

Synthesis of tert-butyl 2-amino-6,7-dihydrothiazolo[5,4-c]pyridine-5(4H)-carboxylate

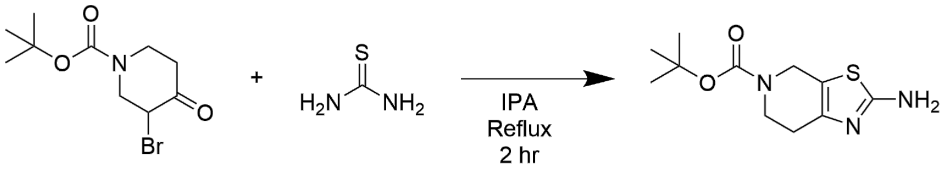

To a 100 mL round bottom flask containing IPA (20 mL), *tert*-butyl 3-bromo-4-oxopiperidine-1-carboxylate (2.00 g, 7.2 mmol, 1.1 equiv) and thiourea (0.500 g, 6.5 mmol, 1 equiv) were added. The mixture was heated to reflux with stirring for 2 hr, and reaction progress monitored via TLC (50% EtOAc in hexanes, visualized with iodine). The resultant mixture was concentrated under reduced pressure to yield the crude product. The crude product was stirred in diethyl ether (30 mL) at room temperature for 10 min to dissolve excess starting material, and the solvent was decanted. This process was then repeated a second time to remove any remaining starting material. The purified product was isolated as a white solid after filtering the mixture with a Büchner funnel (1.80 g, 98% yield).

Synthesis of tert-butyl 2-(thiophene-3-carboxamido)-6,7-dihydrothiazolo[5,4-c]pyridine-5(4H)-carboxylate

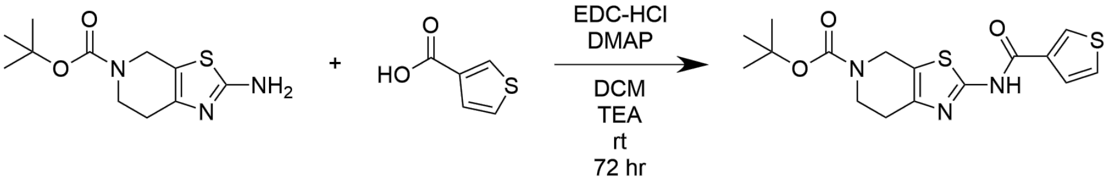

To a flame-dried 250 mL round bottom flask containing DCM (125 mL), *tert*-butyl 2-amino-6,7-dihydrothiazolo[5,4-*c*]pyridine-5(4*H*)-carboxylate (4.23 g, 16.6 mmol, 1 equiv), thiophene-3-carboxylic acid (3.16 g, 16.6 mmol, 1 equiv), EDC-HCl (4.77 g, 24.9 mmol, 1.5 equiv), DMAP (300 mg, 2.46 mmol, 0.15 equiv), and TEA (6.94 mL, 49.8 mmol, 3 equiv) were added. The flask was purged with N_2 and allowed to stir for 72 hr at room temperature under positive N_2 pressure. Thereafter, the DCM was removed *in vacuo*. The crude mixture was resuspended in EtOAc (50 mL) and washed with 2 M NaOH, followed by 1 M HCl, and brine. The organic layers were combined, dried with magnesium sulfate, and concentrated under reduced pressure to yield *tert*-butyl 2-(thiophene-3-carboxamido)-6,7-dihydrothiazolo[5,4-c]pyridine-5(4H)-carboxylate as a pale-yellow solid (4.52 g, 75% yield).

Synthesis of N-(4,5,6,7-tetrahydrothiazolo[5,4-c]pyridin-2-yl)thiophene-3-carboxamide

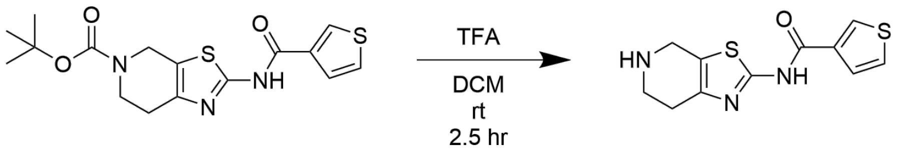

Tert-butyl 2-amino-6,7-dihydrothiazolo[5,4-*c*]pyridine-5(4*H*)-carboxylate (4.41 g, 12 mmol) was dissolved in a 40% TFA in DCM solution (20 mL) in a 100 mL round bottom flask. The reaction was stirred for 2.5 hr at room temperature, and reaction progress was monitored via TLC (50%

EtOAc in hexane). Solvent was removed under reduced pressure, and the remaining solid was resuspended in 2 M NaOH (aq) (50 mL) to remove excess TFA. The product was isolated via simple filtration, and the resuspension/filtration process was repeated 2x to yield *N*-(4,5,6,7-tetrahydrothiazolo[5,4-c]pyridin-2-yl)thiophene-3-carboxamide as a red-beige solid (3.20 g, 100% yield).

Synthesis of tert-butyl (3-chlorophenyl)carbamate

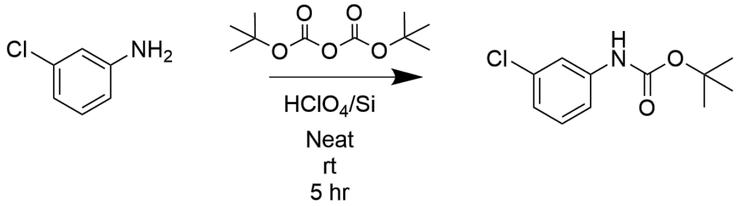

To a 50 mL round bottom flask, 3-chloroaniline (529 mL, 5.0 mmol, 1 equiv) and di-*tert*-butyl dicarbonate (1.20 g, 5.5 mmol, 1.1 equiv) were added. Silica supported perchloric acid (0.35 mmol/g) was then added to the flask, the mixture was stirred for 5 hr at room temperature, and reaction progress was monitored via TLC (10% EtOAc in hexanes). Imidazole (340 mg, 5 mmol, 1 equiv) was added to the reaction mixture and left to stir for 15 min at room temperature. The reaction mixture was diluted into EtOAc (25 mL) and washed with 1 M HCl (3 x 10 mL) and brine (3 x 10 mL). The organic layer was isolated, dried with magnesium sulfate, and concentrated under reduced pressure to yield *tert*-butyl (3-chlorophenyl)carbamate as a white, waxy solid (1.03 g, 91% yield).

Synthesis of N-(3-chlorophenyl)-2-(thiophene-3-carboxamido)-6,7-dihydrothiazolo[5,4-c]pyridine-5(4H)-carboxamide (CP-20)

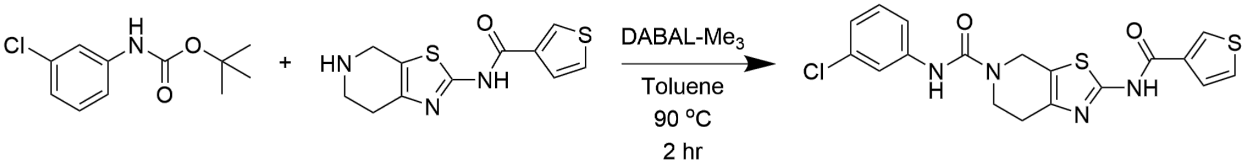

To an oven-dried (100 °C) 25 mL round bottom flask, *N*-(4,5,6,7-tetrahydrothiazolo[5,4-c]pyridin-2-yl)thiophene-3-carboxamide (84.1 mg, 0.41 mmol, 1.1 equiv) in 5 mL toluene was added. DABAL-Me_3 (109 mg, 0.43 mmol, 1.15 equiv) was added, and the mixture was allowed to stir for 20 min at 40 °C. *Tert*-butyl (3-chlorophenyl)carbamate (84.1 mg, 0.37 mmol, 1 equiv) was then added to the flask, the mixture was heated to 90 °C for 2 hr, and reaction progress was monitored via TLC (25% EtOAc in hexane). The reaction was quenched with 1 M HCl (aq.) (4 mL), at which point the product precipitated. The organic layer containing the precipitate was decanted and filtered to obtain *N*-(3-chlorophenyl)-2-(thiophene-3-carboxamido)-6,7-dihydrothiazolo[5,4-*c*]pyridine-5(4*H*)-carboxamide (CP-20) as a beige solid (128 mg, 82% yield).

Additional notes on the synthesis of CP-20 and its derivatives. *Tert*-butyl (3-fluorophenyl)carbamate, *tert*-butyl (3-bromophenyl)carbamate, and *tert*-butyl (3-iodophenyl)carbamate were synthesized following an analogous protocol to that used for the synthesis of *tert*-butyl (3-chlorophenyl)carbamate (varying the halogen on the *meta*-substituted aniline starting material). The identities of all halogen-substituted phenyl carbamate intermediates were confirmed by comparing their NMR spectra with previously reported spectra for these compounds.[1, 2] CP-20-F, CP-20-Br, and CP-20-I were synthesized following an

analogous protocol to that used for the synthesis of the parent compound CP-20, only varying the halogen on the *meta*-substituted phenyl carbamate starting material. All synthetic intermediates had purities of at least 90%, and the final CP-20 derivatives had purities of at least 95%.

Characterization data for synthetic intermediates and CP-20 analogs.

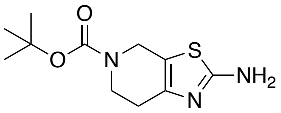

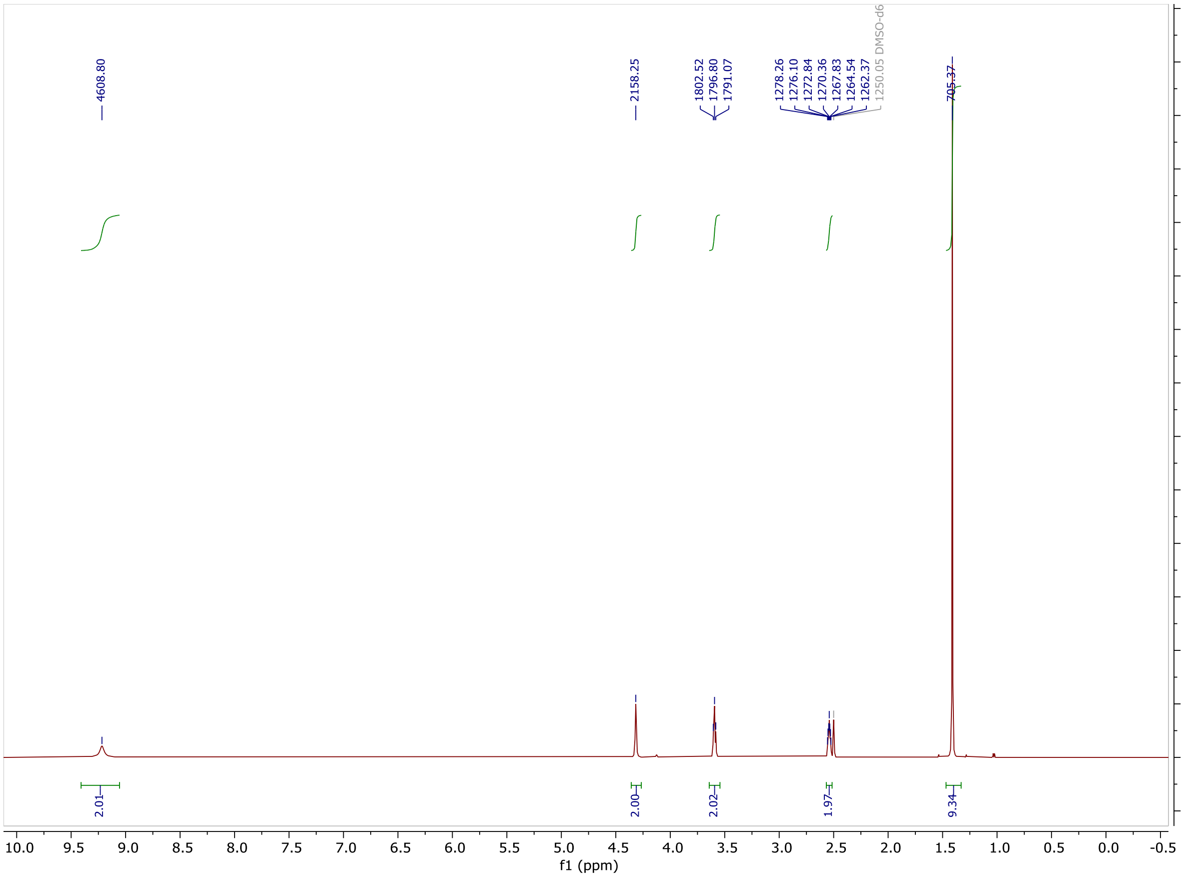

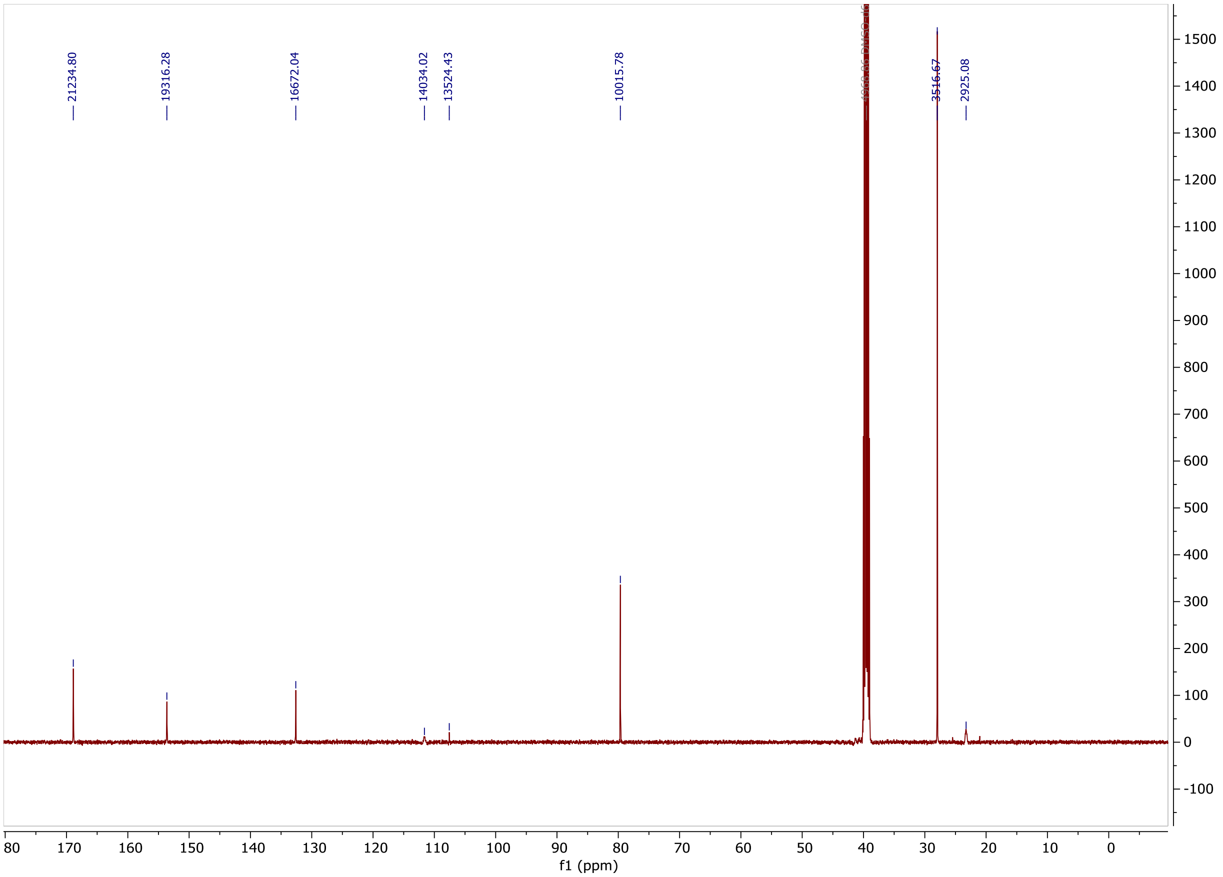

Tert-butyl 2-amino-6,7-dihydrothiazolo[5,4-*c*]pyridine-5(4*H*)-carboxylate: ^{1}H NMR (500 MHz, DMSO-*d*) δ 9.22 (bs, 2H), 4.32 (s, 2H), 3.59 (t, *J* = 5.7 Hz, 2H), 2.54 (m, 2H), 1.41 (s, 9H); ^{13}C NMR (126 MHz, DMSO) δ 168.89, 153.63, 132.60, 111.62, 107.57, 79.66, 27.97, 23.26; ESI-EMM: $[M+H]^{+}$ calculated 256.1114; measured 256.1109.

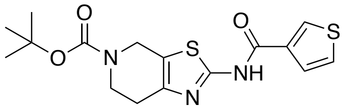

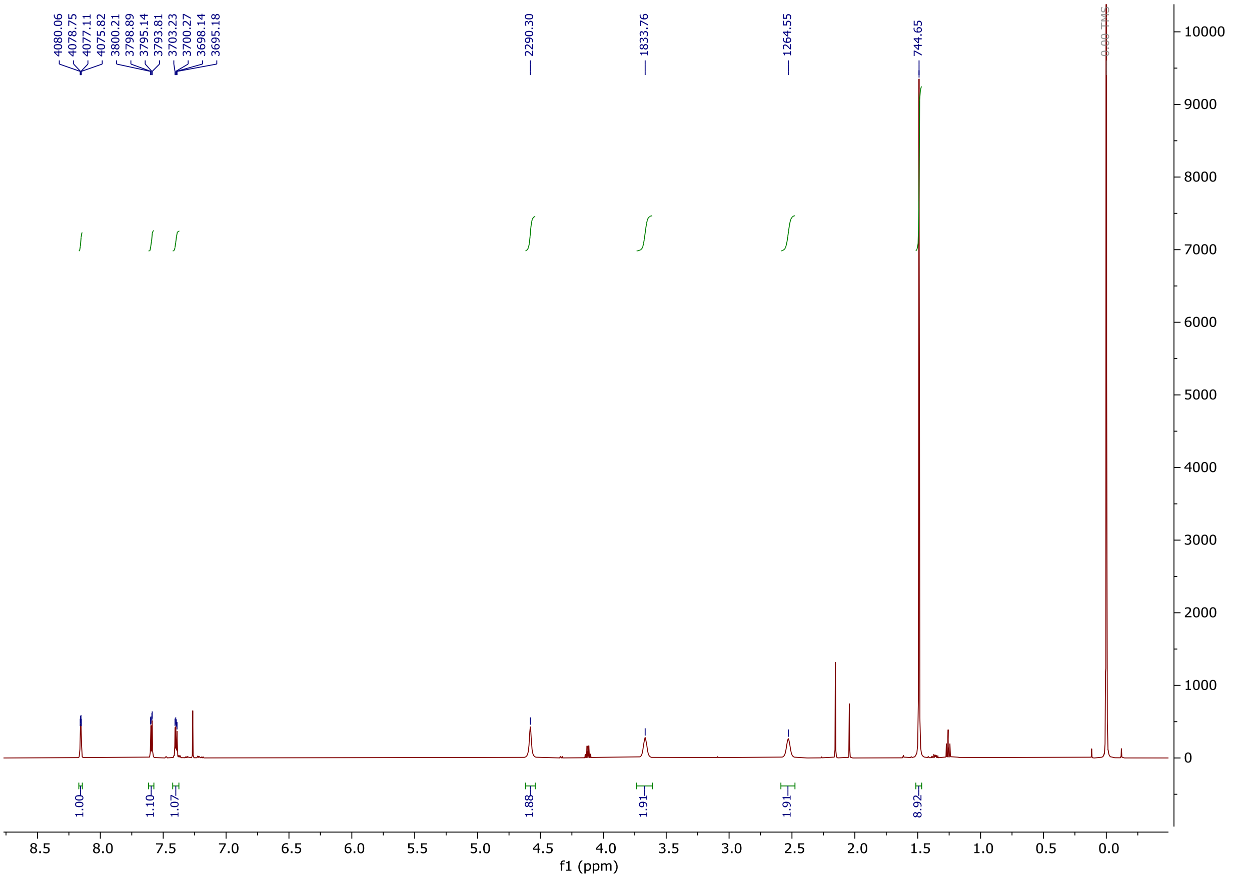
4080.06
4078.75
4077.11
4075.82
3800.21
3798.89
3795.14
3793.81
3703.23
3700.27
3698.14
3695.18
2290.30
1833.76
1264.55
744.65
0.00 TMS
1.00
1.10
1.07
1.88
1.91
1.91
8.92
f1 (ppm)

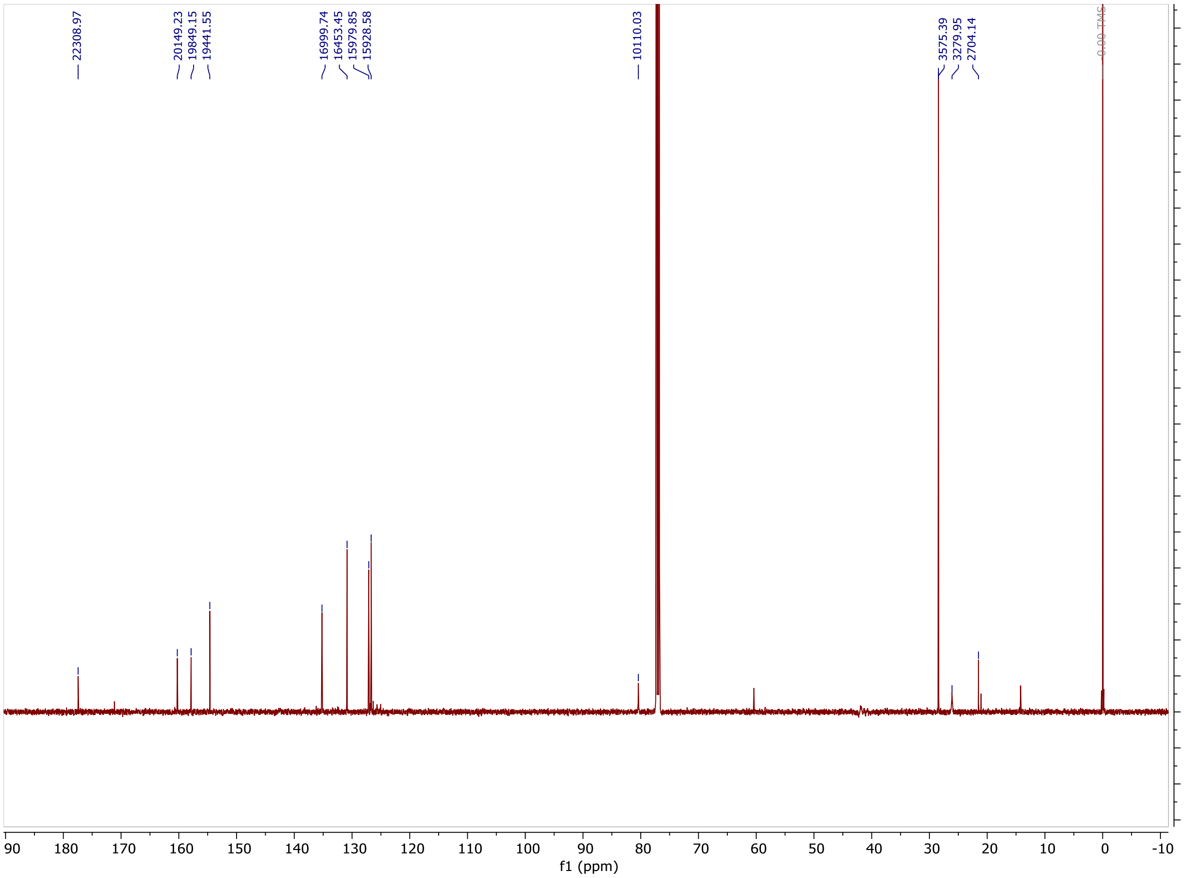

Tert-butyl 2-(thiophene-3-carboxamido)-6,7-dihydrothiazolo[5,4-c]pyridine-5(4H)-carboxylate: ^{1}H NMR (500 MHz, $CDCl_3$) δ 8.16 (dd, *J* = 2.9, 1.3 Hz, 1H), 7.59 (dd, *J* = 5.1, 1.3 Hz, 1H), 7.40 (dd, *J* = 5.1, 3.0 Hz, 1H), 4.58 (s, 2H), 3.67 (s, 2H), 2.53 (s, 2H), 1.49 (s, 9H); ^{13}C NMR (126 MHz, $CDCl_3$) δ 177.44, 160.26, 157.87, 154.63, 135.21, 130.86, 127.10, 126.69, 80.41, 28.44, 26.09, 21.51; ESI-EMM: $[M+H]^+$ calculated 366.0941; measured 366.0936.

[Residual trace solvent/grease peaks (^{1}H NMR, 500 MHz, $CDCl_3$): δ 4.12 (ethyl acetate), 2.15 (acetone), 2.04 (ethyl acetate), 1.26 (ethyl acetate).]

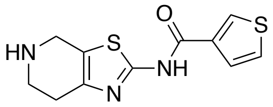

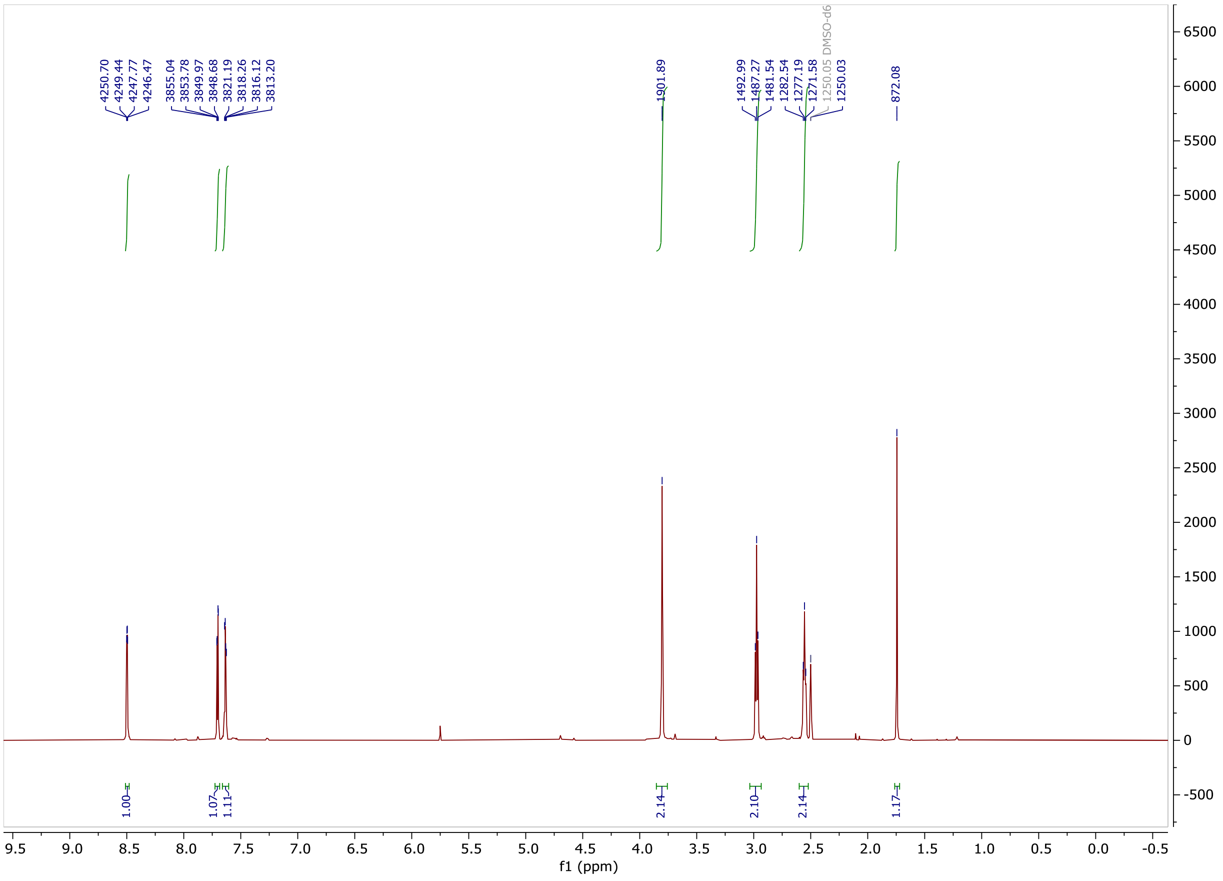
4250.70
4249.44
4247.77
4246.47
3855.04
3853.78
3849.97
3848.68
3821.19
3818.26
3816.12
3813.20
1901.89
1492.99
1487.27
1481.54
1282.54
1277.19
1271.58
1250.05 DMSO-d6
1250.03
872.08
1.00
1.07
1.11
2.14
2.10
2.14
1.17
f1 (ppm)

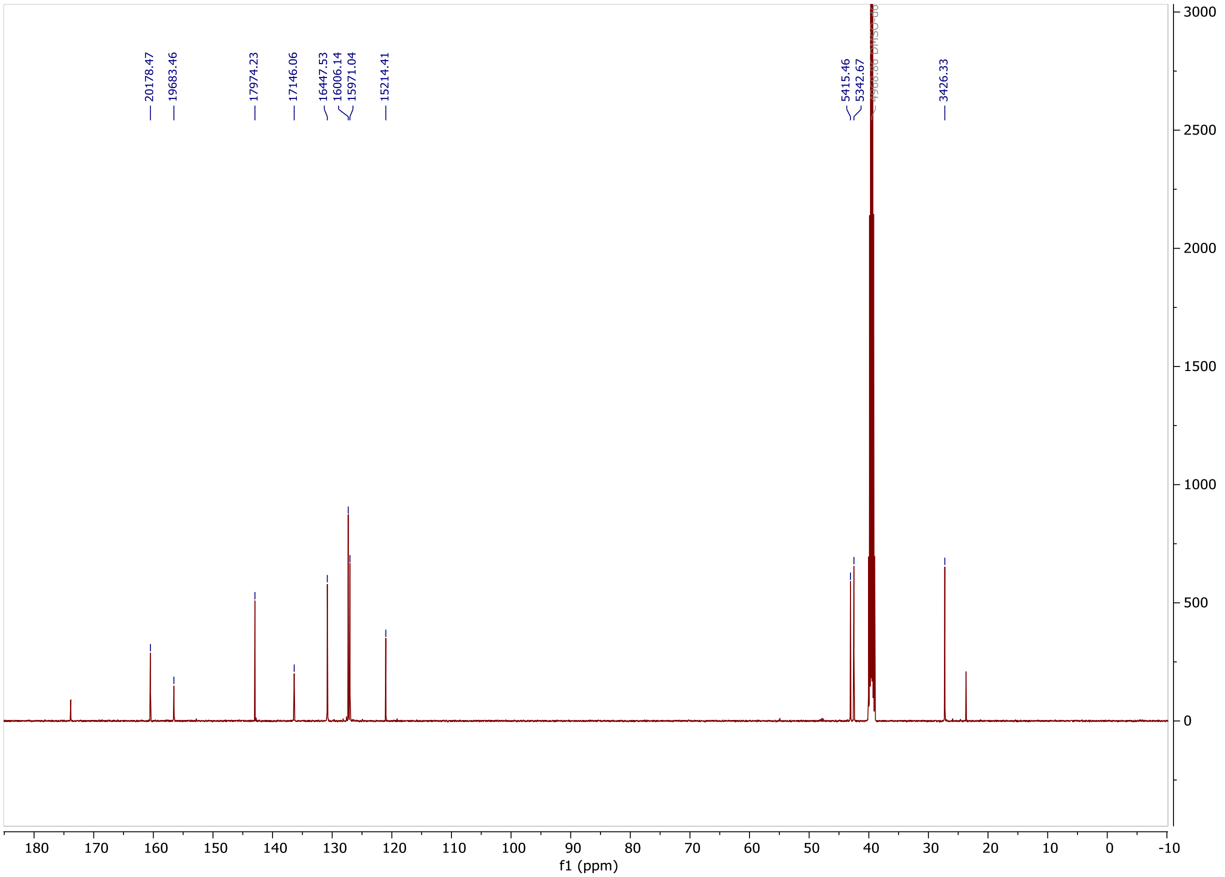

N-(4,5,6,7-tetrahydrothiazolo[5,4-c]pyridine-2-yl)thiophene-3-carboxamide: ^{1}H NMR (500 MHz, DMSO-*d*) δ 8.50 (dd, *J* = 3.0, 1.3 Hz, 1H), 7.70 (dd, *J* = 5.1, 1.3 Hz, 1H), 7.63 (dd, *J* = 5.1, 2.9 Hz, 1H), 3.80 (bs, 2H), 2.97 (t, *J* = 5.7 Hz, 2H), 2.55 (t, *J* = 5.9 Hz, 2H), 1.74 (s, 1H); ^{13}C NMR (126 MHz, DMSO-*d*) δ 160.49, 156.55, 142.96, 136.37, 130.82, 127.31, 127.03, 121.01, 43.07, 42.49, 27.25; ESI-EMM: $[M+H]^+$ calculated 266.0416; measured 266.0414.

[Residual trace solvent/grease peaks (^{1}H NMR, 500 MHz, DMSO-*d*): δ 5.75 (dichloromethane), 2.09 (acetone), 2.07 (acetonitrile), 1.23 (H grease).]

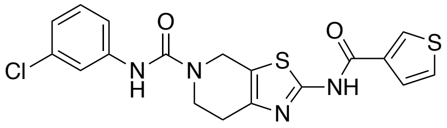
Cl
N
H
O
N
S
N
O
NH
S

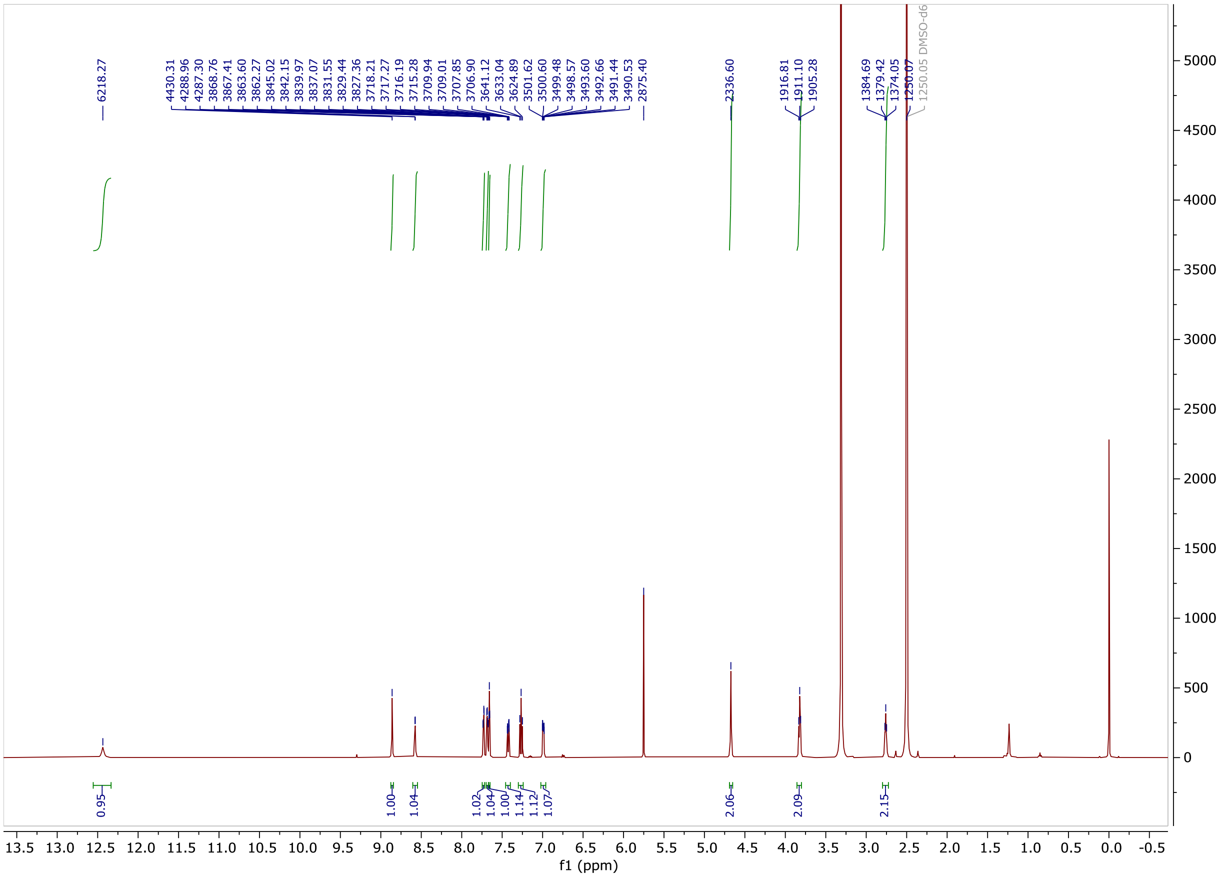
f1 (ppm)
DMSO-d6

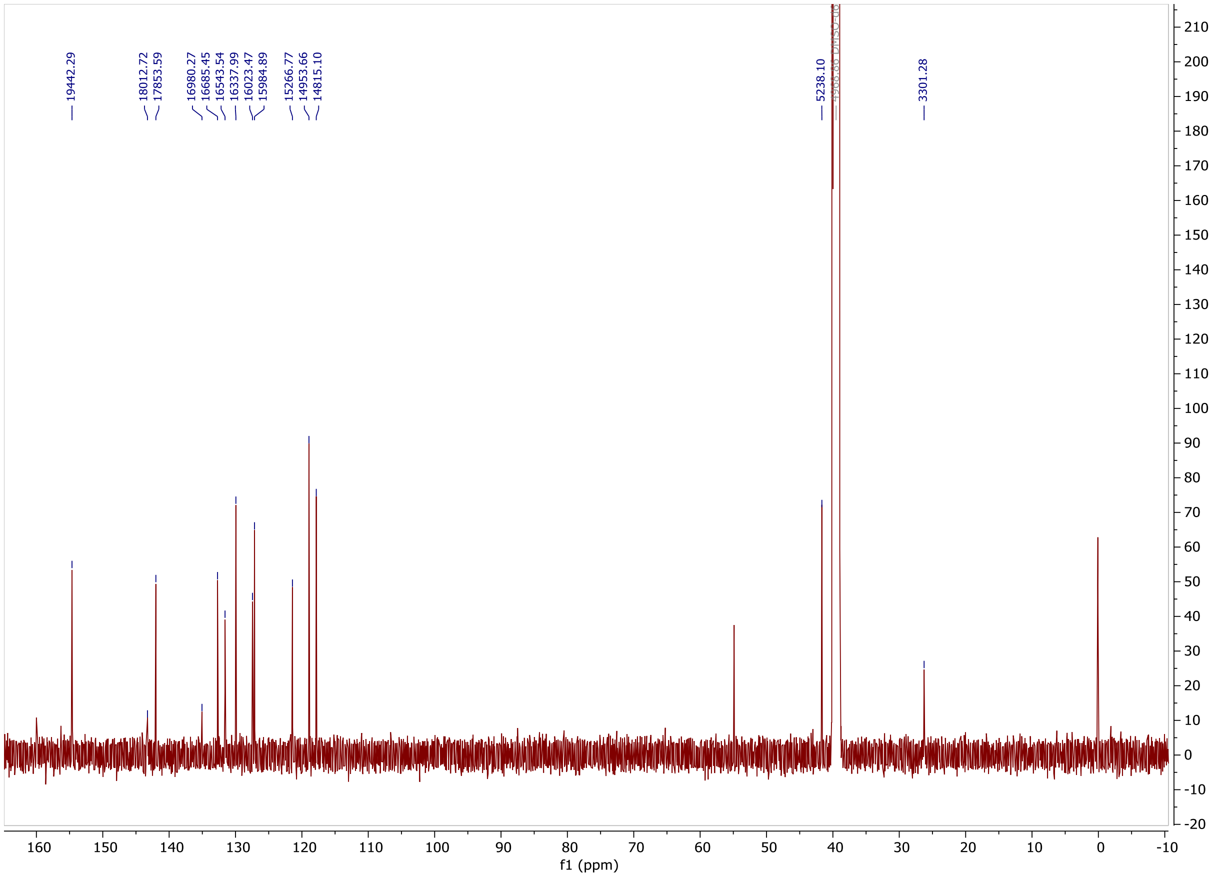

N-(3-chlorophenyl)-2-(thiophene-3-carboxamido)-6,7-dihydrothiazolo[5,4-*c*]pyridine-5(4*H*)-carboxamide (CP-20): ^{1}H NMR (500 MHz, DMSO-*d*) δ 12.44 (s, 1H), 8.86 (s, 1H), 8.58 (d, *J* = 1.7 Hz, 1H), 7.73 (dd, *J* = 5.1, 1.3 Hz, 1H), 7.68 (dd, *J* = 5.1, 2.9 Hz, 1H), 7.66 (t, *J* = 2.1 Hz, 1H), 7.42 (ddd, *J* = 8.4, 2.1, 0.9 Hz, 1H), 7.27 (t, *J* = 8.1 Hz, 1H), 6.99 (ddd, *J* = 8.0, 2.1, 1.0 Hz, 1H), 4.67 (s, 2H), 3.82 (t, *J* = 5.8 Hz, 2H), 2.76 (t, *J* = 5.3 Hz, 2H); ^{13}C NMR (126 MHz, DMSO) δ 159.98, 154.64, 142.00, 132.71, 131.58, 129.94, 127.44, 127.14, 121.42, 118.93, 117.83, 41.66 (2C), 26.26; ESI-EMM: $[M+H]^{+}$ calculated 419.0398; measured 419.0394.

[Residual trace solvent/grease peaks (^{1}H NMR, 500 MHz, DMSO-*d*): δ 5.75 (dichloromethane), 3.31 (water), 1.23 (H grease), 0.85 (H grease).]

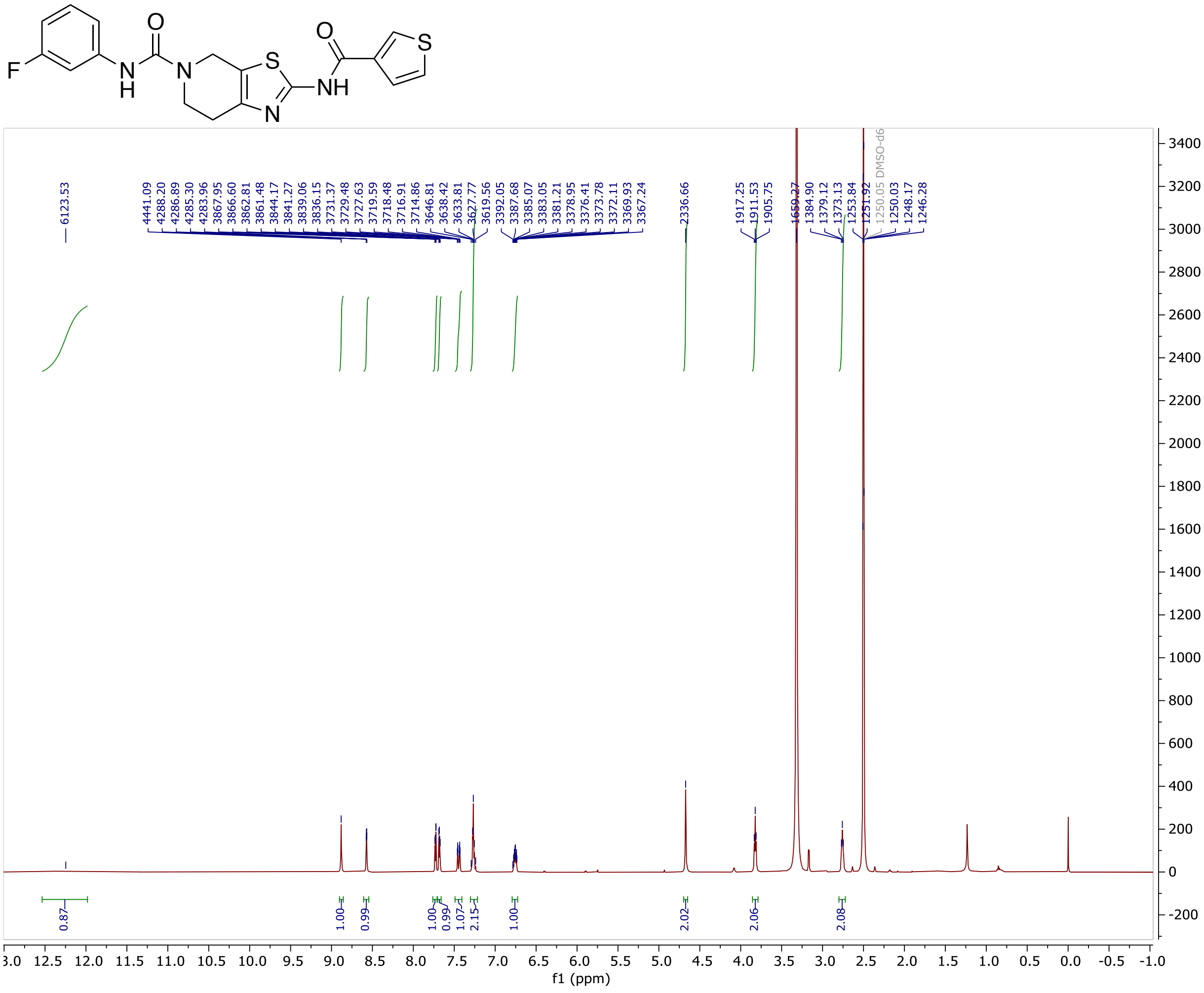

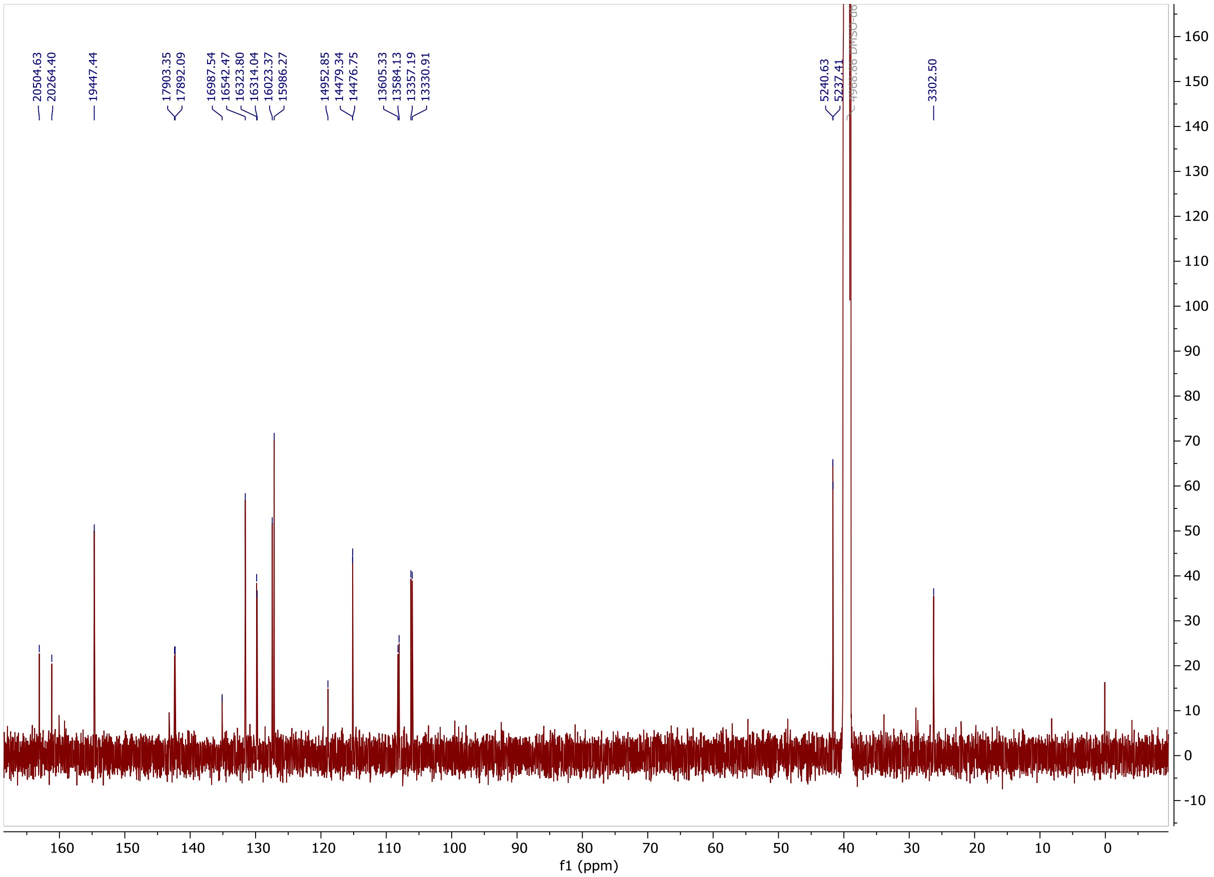

N-(3-fluorophenyl)-2-(thiophene-3-carboxamido)-6,7-dihydrothiazolo[5,4-*c*]pyridine-5(4*H*)-carboxamide (CP-20-F): ^{1}H NMR (500 MHz, DMSO-*d*) δ 12.25 (s, 1H), 8.88 (s, 1H), 8.57 (dd, *J* = 2.9, 1.3 Hz, 1H), 7.73 (dd, *J* = 5.1, 1.3 Hz, 1H), 7.68 (dd, *J* = 5.1, 2.9 Hz, 1H), 7.45 (ddd, *J* = 8.4, 2.1, 0.9 Hz, 1H), 7.31 – 7.22 (m, 2H), 6.76 (m, 1H), 4.67 (s, 2H), 3.82 (t, *J* = 5.8 Hz, 2H), 2.76 (t, *J* = 5.9 Hz, 1H); ^{13}C NMR (126 MHz, DMSO-*d*) δ 162.13 (d, *J* = 240.2 Hz), 154.68, 142.35 (d, *J* = 11.3 Hz), 135.11, 131.57, 129.79 (d, *J* = 9.8 Hz), 127.44, 127.15, 118.93, 115.15 (d, *J* = 2.6 Hz), 108.13 (d, *J* = 21.2 Hz), 106.13 (d, *J* = 26.3 Hz), 41.68, 41.66, 26.27; ESI-EMM: $[M+H]^{+}$ calculated 403.0693; measured 403.0687.

[Residual trace solvent/grease peaks (^{1}H NMR, 500 MHz, DMSO-*d*): δ 3.32 (water), 1.23 (H grease), 0.84 (H grease).]

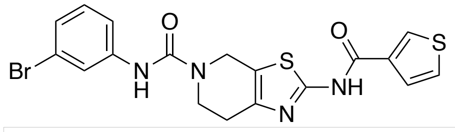
O
Br
N
H
N
S
N
NH
O
S

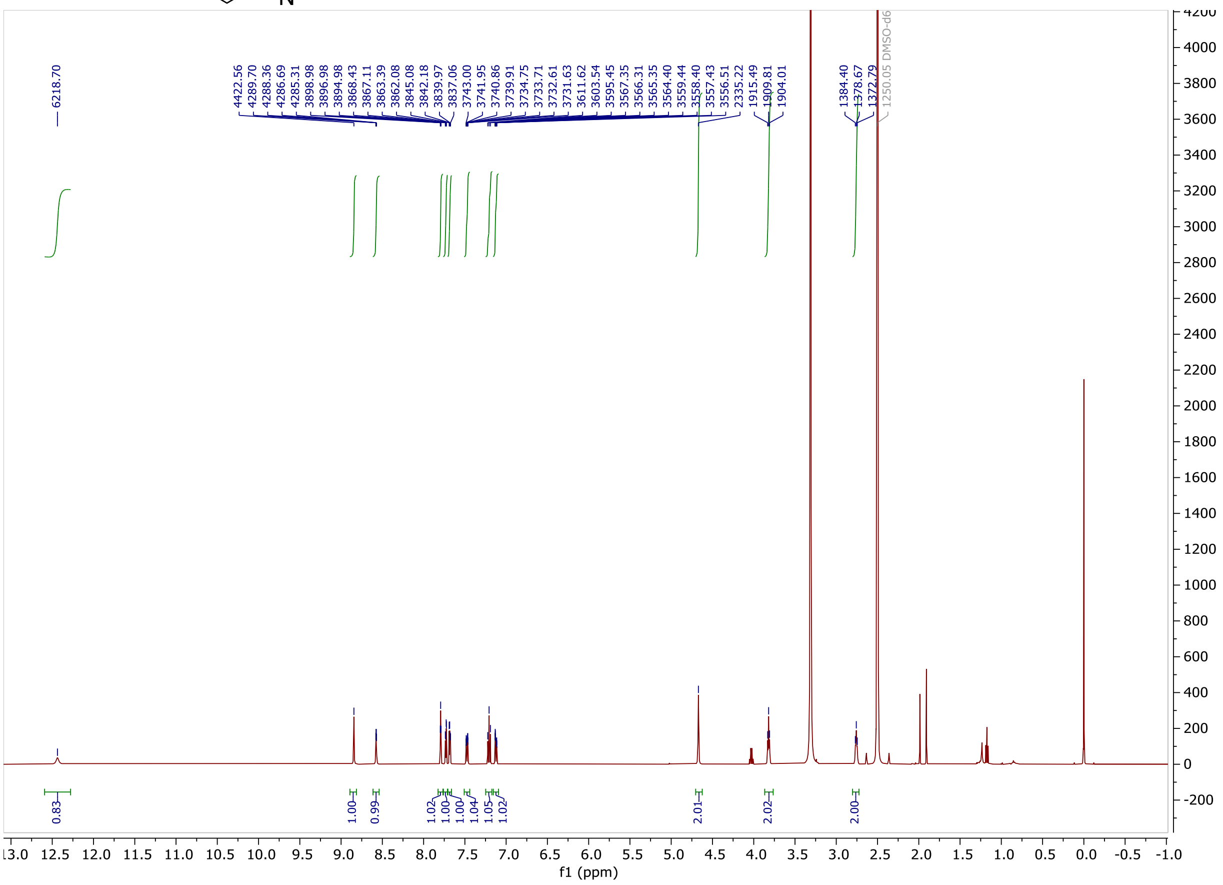
6218.70
4422.56
4289.70
4288.36
4286.69
4285.31
3898.98
3896.98
3894.98
3868.43
3867.11
3863.39
3862.08
3845.08
3842.18
3839.97
3837.06
3743.00
3741.95
3740.86
3739.91
3734.75
3733.71
3732.61
3731.63
3611.62
3603.54
3595.45
3567.35
3566.31
3565.35
3564.40
3559.44
3558.40
3557.43
3556.51
2335.22
1915.49
1909.81
1904.01
1384.40
1378.67
1372.79
1250.05 DMSO-d6
0.83
1.00
0.99
1.02
1.00
1.00
1.04
1.05
1.02
2.01
2.02
2.00
f1 (ppm)

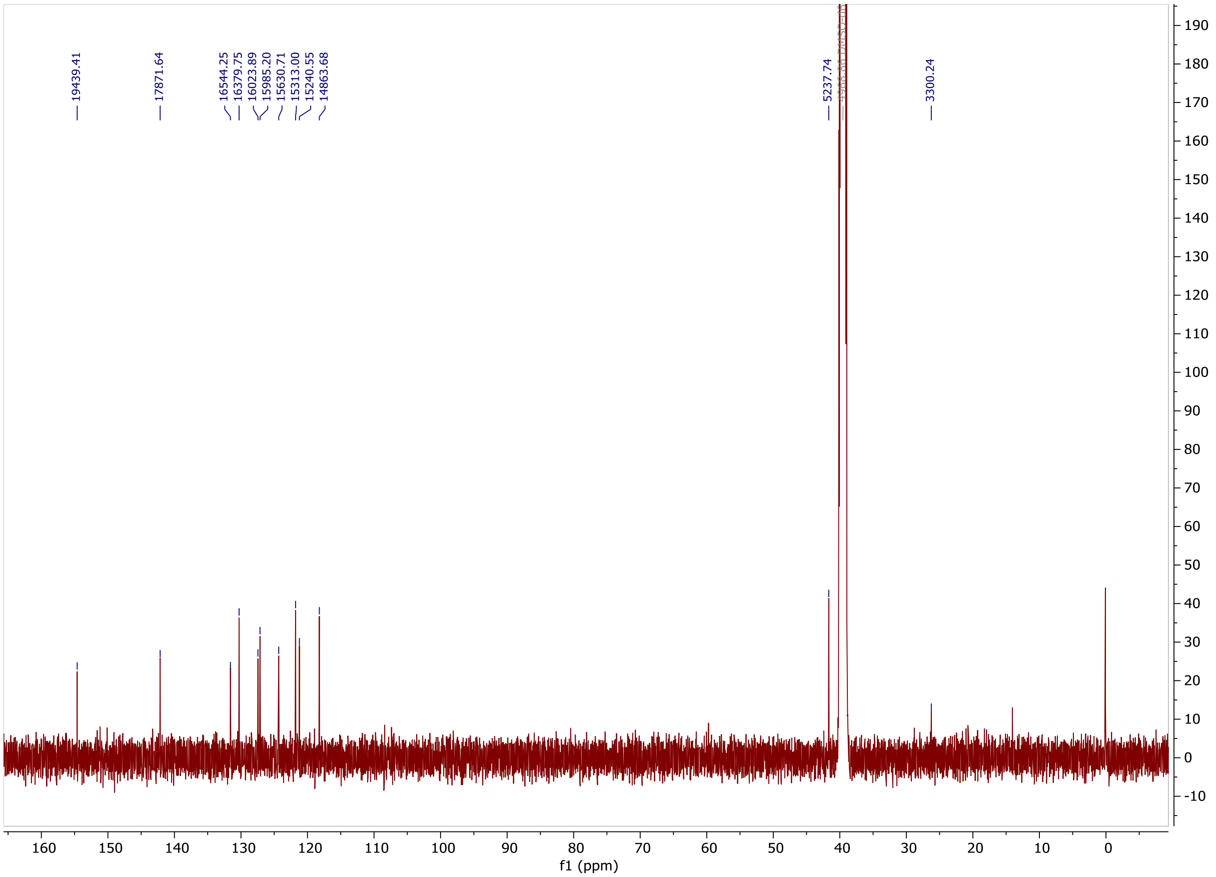

N-(3-bromophenyl)-2-(thiophene-3-carboxamido)-6,7-dihydrothiazolo[5,4-*c*]pyridine-5(4*H*)-carboxamide (CP-20-Br): ^{1}H NMR (500 MHz, DMSO-*d*) δ 12.44 (s, 1H), 8.84 (s, 1H), 8.57 (dd, *J* = 3.0, 1.4 Hz, 1H), 7.79 (t, *J* = 2.0 Hz, 1H), 7.73 (dd, *J* = 5.0, 1.3 Hz, 1H), 7.68 (dd, *J* = 5.1, 2.9 Hz, 1H), 7.47 (ddd, *J* = 8.3, 2.1, 1.0 Hz, 1H), 7.21 (t, *J* = 8.1 Hz, 1H), 7.12 (ddd, *J* = 7.9, 1.9, 1.0 Hz, 1H), 4.67 (s, 2H), 3.82 (t, *J* = 5.7 Hz, 2H), 2.76 (t, *J* = 5.8 Hz, 2H); ^{13}C NMR (126 MHz, DMSO-*d*) δ 154.61, 142.14, 131.59, 130.28, 127.45, 127.14, 124.32, 121.79, 121.22, 118.22, 41.66 (2C), 26.25; ESI-EMM: [M+H]$^{+}$ calculated 462.9893; measured 462.9886.

[Residual trace solvent/grease peaks (^{1}H NMR, 500 MHz, DMSO-*d*): δ 4.03 (ethyl acetate), 3.31 (water), 1.99 (ethyl acetate), 1.91 (acetic acid), 1.23 (H grease), 1.17 (ethyl acetate), 0.91–0.78 (H grease).]

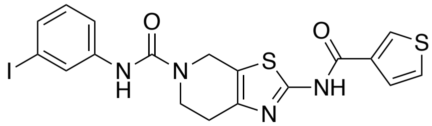

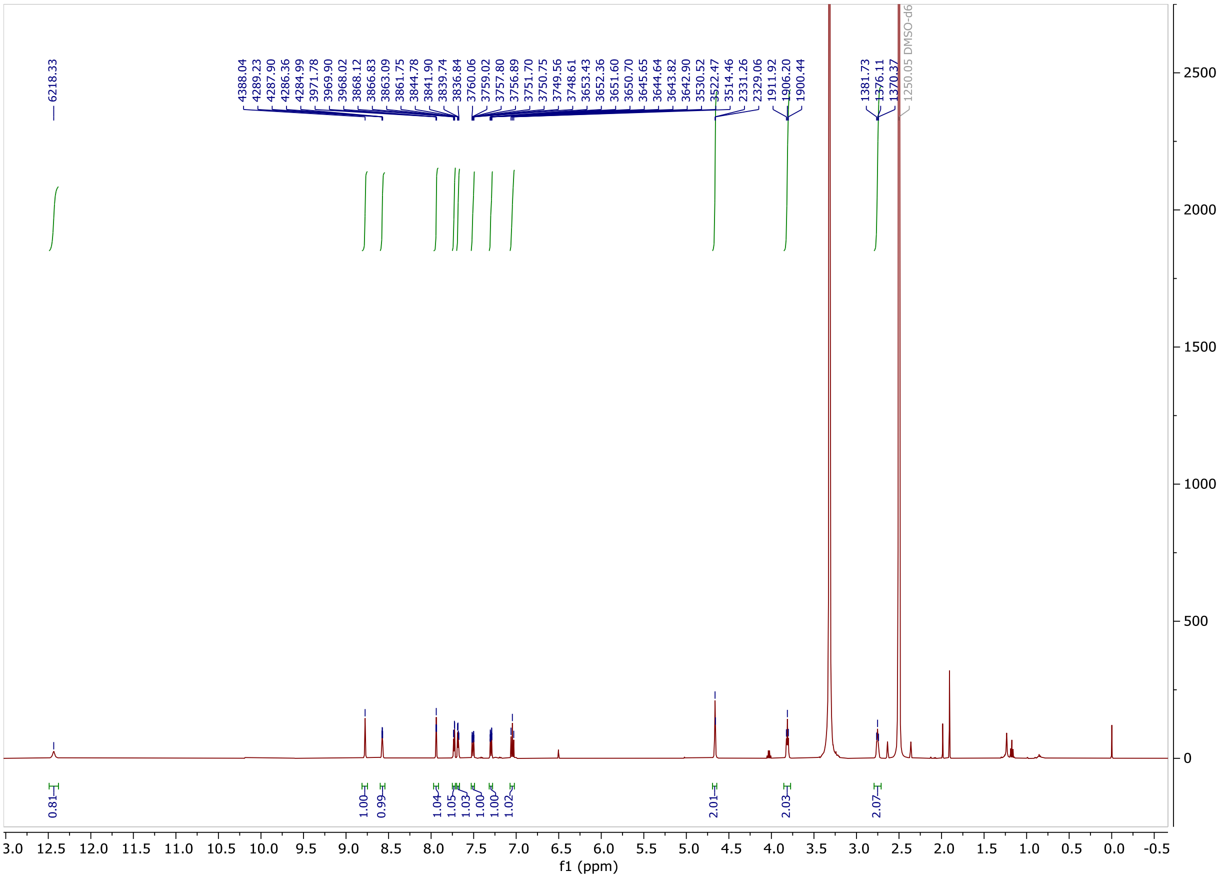

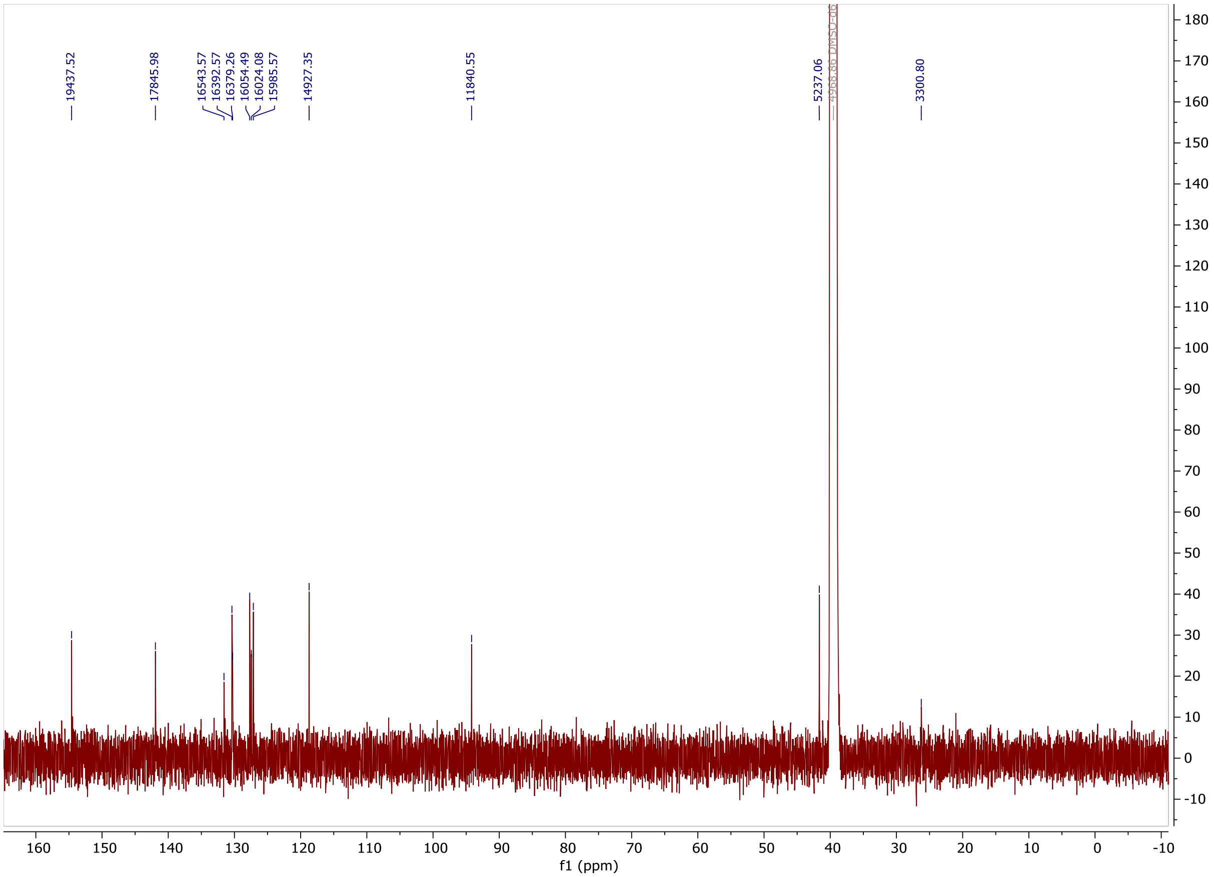

N-(3-iodophenyl)-2-(thiophene-3-carboxamido)-6,7-dihydrothiazolo[5,4-*c*]pyridine-5(4*H*)-carboxamide (CP-20-I): ^{1}H NMR (500 MHz, DMSO-*d*) δ 12.44 (s, 1H), 8.78 (s, 1H), 8.57 (dd, *J* = 2.9, 1.3 Hz, 1H), 7.94 (t, *J* = 1.9 Hz, 1H), 7.73 (dd, *J* = 5.1, 1.3 Hz, 1H), 7.68 (dd, *J* = 5.0, 2.9 Hz, 1H), 7.51 (ddd, *J* = 8.3, 2.2, 1.0 Hz, 1H), 7.30 (ddd, *J* = 7.8, 1.8, 1.0 Hz, 1H), 7.04 (t, *J* = 8.0 Hz, 1H), 4.66 (s, 2H), 3.81 (t, *J* = 5.7 Hz, 2H), 2.75 (t, *J* = 5.7 Hz, 2H); ^{13}C NMR (126 MHz, DMSO-*d*) δ 154.60, 141.94, 131.58, 130.38, 130.27, 127.69, 127.45, 127.14, 118.73, 94.17, 41.65 (2C), 26.25; ESI-EMM: $[M+H]^{+}$ calculated 510.9754; measured 510.9749.

[Residual trace solvent/grease peaks (^{1}H NMR, 500 MHz, DMSO-*d*): δ 4.03 (ethyl acetate), 3.32 (water), 1.99 (ethyl acetate), 1.91 (acetic acid), 1.23 (H grease), 1.17 (ethyl acetate), 0.89–0.77 (H grease).]

<u>Synthesis of peptide probes and compound characterization data.</u>

Peptide synthesis. _S. aureus_ AIP-I and AIP-III D4A were synthesized according to our reported methods for Dawson Dbz AM resin-based AIP synthesis[3] and acquired from in-house stocks. TAMRA-AIP-III D4A was synthesized according to our previously reported methods for Dawson Dbz AM resin-based AIP synthesis[3] (on 30 mg resin) with the following modifications: the final N-terminal amino acid residue (Ile) contained a 9-fluorenylmethoxycarbonyl (Fmoc)-protected backbone amine. The Fmoc group was removed using a 20% piperidine solution in DMF (2 mL, 3 x 10 min at room temperature). To couple 5(6)-carboxytetramethylrhodamine (TAMRA) to the newly liberated N-terminus, a solution of TAMRA (4 equiv), HBTU (4 equiv), and DIPEA (8 equiv) in DMF (2 mL) were added to the reaction tube containing resin-bound peptide and incubated with shaking at room temperature overnight in the dark. Resin manipulation, peptide cleavage/deprotection, macrocyclization, and purification via RP-HPLC were conducted as described previously.[4] An HPLC trace (UV detection at 220 nm) and MS data for TAMRA-AIP-III D4A are provided below. TAMRA-AIP-III D4A was prepared for use as a 1 mM stock solution in DMSO and stored in the dark at -20 °C.

Characterization data for TAMRA-AIP-III D4A.

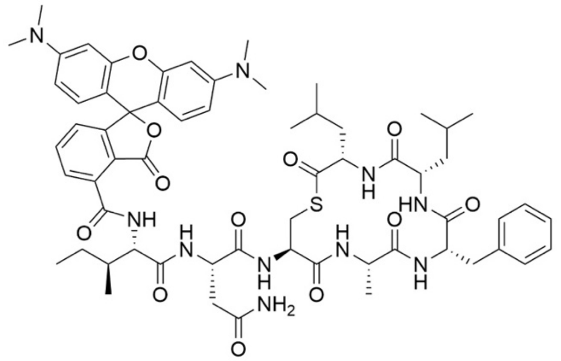

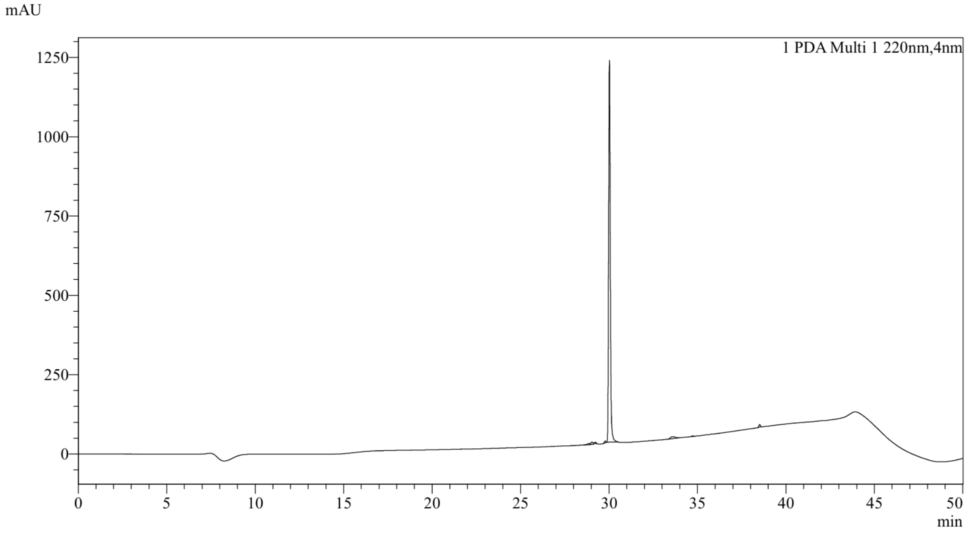

Purity: 96% (220 nm); ESI-EMM: $[M+H]^+$ calculated 1187.5594; measured 1187.5586.

General biological reagents and methods.

Reagents. Water (18 MΩ) was purified by using an Arium Pro ultrapure water system (Sartorius). Dimethyl sulfoxide (DMSO), sodium sulfate, sodium chloride, glucose, protease inhibitor cocktail, imidazole, yeast extract, sodium hydrogen phosphate, and magnesium chloride were purchased from Sigma-Aldrich. Nitrocefin was purchased from Abcam. Sodium azide was purchased from Acros Organics. Tris(2-carboxyethyl)phosphine hydrochloride (TCEP) and isopropyl β-D-1-thiogalactopyranoside (IPTG) were purchased from Gold Biotechnology. Fos-choline-12, Fos-choline-14, 1-palmitoyl-2-oleoyl-glycero-3-phosphocholine (POPC), and 1-palmitoyl-2-oleoyl-sn-glycero-3-phosphoglycerol (POPG) were purchased from Anatrace. His_7-TEV protease was purified according to previously reported methods and acquired from in-house stocks.[5] Ni-NTA resin and Triton X-100 were purchased from Thermo-Fisher. Tryptone and Bio-Safe-II liquid scintillation counting cocktail were purchased from RPI. Sodium cholate hydrate and ATP were purchased from Chem Impex. Tris and 4-(2-hydroxyethyl)-1-piperazineethanesulfonic acid (HEPES) were purchased from DOT Scientific. Bio-Beads and pre-cast polyacrylamide gels (10%) for SDS-PAGE analysis were purchased from Bio-Rad. ^{32}P-labeled ATP was purchased from Perkin Elmer.

Bacterial growth conditions. The strains and plasmids used in this study are listed in **Table S1**. *Staphylococcus aureus* fluorescent reporter strains (AH1677, AH430, AH1747, and AH1872) and *S. aureus* RN9222 were grown in Brain Heart Infusion broth (BHI, Teknova). *Staphylococcus epidermidis* fluorescent reporter strains (AH3408 and AH3623), *S. aureus* AH3048, and *S. aureus* USA300 LAC were grown in NutriSelect Basic Tryptic Soy Broth (TSB, Sigma-Aldrich). *Escherichia coli* strains were grown in Luria-Bertani media (LB, Sigma-Aldrich) or Terrific Broth (TB, Sigma-Aldrich), as specified below.

Instrumentation. Bacterial cultures were grown in a standard laboratory incubator heated to 37 °C with shaking at 200 rpm unless otherwise specified. Protein UV absorbance readings were measured using a NanoDrop 2000c spectrophotometer (Thermo-Fisher). All experiments requiring a plate reader were conducted using a Biotek Synergy 2 plate reader or Perkin Elmer EnVision plate reader as specified below. Size exclusion chromatography (SEC) was performed using an AKTA Purifier Fast Protein Liquid Chromatograph (FPLC) (GE Healthcare) running Unicorn v5 software.

Table S1. Bacterial strains and plasmids used in this study.

Strain	Description[a]	References
S. aureus USA300 LAC	*agr*-I	In-house stock, sourced from A. Horswill
S. aureus AH1677	*agr*-I<br>USA300 LAC / pDB59 (Cam^r)	Kirchdoerfer *et al.*, 2011[6]<br>Yarwood *et al.*, 2003[7]
S. aureus AH430	*agr*-II<br>SA502A / pDB59 (Cam^r)	Malone *et al.*, 2007[8]<br>Yarwood *et al.*, 2003[7]
S. aureus AH1747	*agr*-III<br>MW2 / pDB59 (Cam^r)	Kirchdoerfer *et al.*, 2011[6]<br>Yarwood *et al.*, 2003[7]
S. aureus AH1872	*agr*-IV<br>MN EV / pDB59 (Cam^r)	Kirchdoerfer *et al.*, 2011[6]<br>Yarwood *et al.*, 2003[7]
S. aureus RN9222	RN6911 / pRN7062 (Erm^r)	Lyon *et al.*, 2000[9]
S. aureus AH3048	ROJ48 / pCM63 (Cam^r)	Sully *et al.*, 2014[10]
S. epidermidis AH3408	*agr*-I<br>ATCC 12228 / pCM40 (Erm^r)	Olson *et al.*, 2014[11]
S. epidermidis AH3623	*agr*-II<br>1457 *ica::dhfr* / pCM40 (Erm^r)	Olson *et al.*, 2014[11]
E. coli C43 (DE3)<br>pBW-C1-1	pET24b encoding *agrC-I-His$_6$* for the expression of AgrC-I (Kan^r)	Wang *et al.*, 2014[12]
E. coli BL21 (DE3)<br>pMSP1E3D1	pET28a encoding *His$_7$-TEV-MSP1E3D1* for the expression of MSP1E3D1 (Kan^r)	Ritchie *et al.*, 2009[13]

[a]Erm^r = erythromycin resistance; Cam^r = chloramphenicol resistance; Kan^r = kanamycin resistance.

<u>**Bacterial reporter assay methods.**</u>

***S. aureus agr* fluorescence reporter assays.** Overnight cultures of selected *S. aureus agr* reporter strains (*S. aureus* strains AH1677, AH430, AH1747, AH1872) were grown in BHI medium (2 mL) supplemented with chloramphenicol (10 μg/mL) for 20 hr at 37 °C with shaking at 200 rpm. These cultures were diluted 1:50 in fresh, antibiotic-free BHI medium to generate inoculating cultures. Aliquots (2 μL) of compounds (derived from stock solutions in DMSO) were

added to the wells of black 96-well microtiter plates (Corning) in a 3-fold dilution series. DMSO (2 µL) was plated as a vehicle control. Inoculating culture (198 µL/well) was added to wells containing compounds or vehicle control. Fresh BHI medium (200 µL) was used as a sterility and fluorescence background control. Plates were incubated for 24 hr at 37 °C with shaking at 700 rpm in a Stuart SI505 microtiter plate shaker incubator. Fluorescence (500 nm excitation, 540 nm emission) and OD_{600} measurements were obtained using a Biotek Synergy 2 plate reader running Gen 5 software (version 1.05). Fluorescence measurements were corrected to media background fluorescence (i.e., media fluorescence set to 0%) and normalized to OD_{600} and vehicle control (i.e., vehicle fluorescence set to 100%). Dose-response curves, maximum inhibition values, and potency values were generated using four-parameter non-linear regression analysis ([Inhibitor] vs. response) in GraphPad Prism software (version 10.3.0). Three biological replicates were collected for each experimental condition.

***S. epidermidis agr* fluorescence reporter assays.** Overnight cultures of selected *S. epidermidis agr* reporter strains (*S. epidermidis* strains AH3408 and AH3623) were grown in TSB medium (3 mL) supplemented with erythromycin (10 µg/mL) for 24 hr at 37 °C with shaking at 200 rpm. These cultures were diluted 1:50 in fresh, antibiotic-free TSB medium to generate inoculating cultures. Aliquots (2 µL) of compounds (derived from stock solutions in DMSO) were added to the wells of black 96-well microtiter plates (Corning) in a 3-fold dilution. DMSO (2 µL) was plated as a vehicle control. Inoculating culture (198 µL/well) was added to wells containing compounds or vehicle control. Fresh TSB medium (200 µL) was used as a sterility and fluorescence background control. Plates were incubated for 24 hr at 37 °C with shaking at 700 rpm in a Stuart SI505 microtiter plate shaker incubator. Fluorescence (500 nm excitation, 540 nm emission) and OD_{600} measurements were obtained using a Biotek Synergy 2 plate reader running Gen 5 software (version 1.05). Fluorescence measurements were corrected to media background fluorescence (i.e., media fluorescence set to 0%) and normalized to OD_{600} and vehicle control (i.e., vehicle fluorescence set to 100%). Dose-response curves, maximum inhibition/activation values, and potency values were generated using four-parameter non-linear regression analysis ([Inhibitor] vs. response or [Agonist] vs. response) in GraphPad Prism software (version 10.3.0). Three biological replicates were collected for each experimental condition.

S. aureus* β-lactamase reporter assays.* The β-lactamase assay protocol closely followed that of Tal-Gan et al.,[14] with some small modifications. Antagonism of AgrCA was measured using the *S. aureus* β-lactamase reporter strain RN9222. Overnight cultures of *S. aureus* RN9222 were grown in BHI medium (3 mL) supplemented with erythromycin (10 µg/mL) for 16 hr at 37 °C with shaking at 200 rpm. These cultures were diluted 1:50 in fresh, antibiotic-free BHI medium and further incubated at 37 °C with 200 rpm shaking until reaching an OD_{600} of approximately 0.16. Aliquots (1 µL) of inhibitors (derived from stock solutions in DMSO) were added to the wells of clear 96-well microtiter plates (Corning) in a 3-fold dilution series. To each well containing inhibitor, along with a separate positive control well, an aliquot (1 µL) of *S. aureus* AIP-I (stock concentration of 10 µM) was added (to yield a final concentration of 100 nM). DMSO (2 µL) was plated as a negative control for β-lactamase activity. All wells were adjusted to achieve a final concentration of DMSO of 2% (v/v). Inoculating culture (98 µL/well) was added to wells containing compounds or vehicle control. Fresh BHI medium (100 µL) was used as a sterility control. Plates were incubated for 1 hr at 37 °C with shaking at 250 rpm in a Stuart SI505 microtiter plate shaker incubator. Following incubation, the OD_{600} of each well was measured using a Biotek Synergy 2 plate reader running Gen 5 software (version 1.05). Aliquots (55 µL/well) of a solution of sodium azide (4.55 mM) and nitrocefin (120 µg/mL) were added to each well containing culture. Plates were incubated for 20 min in the dark at room temperature. Absorbance at 495 nm was measured using a Biotek Synergy 2 plate reader. Absorbance

readings were corrected to the negative control absorbance (i.e., DMSO absorbance set to 0%) and normalized to OD_{600} and the positive control absorbance (i.e., AIP-I absorbance set to 100%), with percentage values denoting relative β-lactamase activities. Dose-response curves, maximum inhibition values, and potency values were generated using four-parameter non-linear regression analysis ([Inhibitor] vs. response) in GraphPad Prism software (version 10.3.0). Three biological replicates were collected for each experimental condition.

AgrA luminescence reporter assays. Overnight cultures of *S. aureus* AH3048 were grown in TSB medium (3 mL) supplemented with chloramphenicol (10 μg/mL) for 16 hr at 37 °C with shaking at 200 rpm. These cultures were diluted 1:500 in fresh, antibiotic-free TSB medium containing 0.25% (w/v) xylose to form inoculating cultures. Aliquots (2 μL) of compounds (derived from stock solutions in DMSO) were added to the wells of white 96-well microtiter plates (Corning) in a 3-fold dilution series. DMSO (2 μL) was plated as a vehicle control. Inoculating culture (198 μL/well) was added to wells containing compounds or vehicle control. Fresh TSB medium (200 μL) was used as a sterility and fluorescence background control. Plates were incubated for 6 hr at 37 °C with shaking at 700 rpm in a Stuart SI505 microtiter plate shaker incubator. Luminescence and OD_{600} measurements were taken with a Biotek Synergy 2 plate reader running Gen 5 software (version 1.05). Luminescence measurements were corrected to media background luminescence (i.e., media luminescence set to 0%) and normalized to OD_{600} and vehicle control (i.e., vehicle luminescence set to 100%). Dose-response curves, maximum inhibition values, and potency values were generated using four-parameter non-linear regression analysis ([Inhibitor] vs. response) in GraphPad Prism software (version 10.3.0). Three biological replicates were collected for each experimental condition.

Protein production and *in vitro* assay methods.

Recombinant expression and purification of AgrC-I. Recombinant AgrC-I-His_6 was expressed in *E. coli* and purified based on methods developed by Muir and co-workers with several modifications.[12, 15] *E. coli* C43 (DE3) (harboring the pBW-C1-1 plasmid for AgrC-I-His_6 expression) culture was prepared in aqueous LB medium (10 mL) containing kanamycin (50 μg/mL) and incubated at 37 °C with shaking at 200 rpm overnight. Two flasks of LB culture medium (500 mL per flask) were supplemented with glucose (2 g per flask) and sodium sulfate (120 mg per flask) and sterilized. To each flask, an aliquot of overnight *E. coli* culture (2 mL) was added, forming expression cultures. The expression cultures were incubated at 37 °C with shaking at 200 rpm until reaching an OD_{600} of 0.5 (approximately 4 hr). Once the OD_{600} was reached, protein expression was induced by adding IPTG (final concentration of 0.5 mM) to each culture. The cultures were then incubated at 22 °C with shaking at 220 rpm for 16 hr.

E. coli cells were pelleted by centrifugation at 5000 x g for 20 min at 22 °C. Cell pellets were resuspended in TBS buffer (20 mM Tris, 100 mM NaCl, 1 mM TCEP, 1x protease inhibitor, pH 8) at 4 °C at a ratio of 5 mL buffer per 1 g of pellet. Cell membranes were disrupted via sonication (35% amplitude, 4 sec on followed by 1 sec off for 12 min). Cell debris was then removed by centrifuging the lysate at 22600 x g for 20 min at 4 °C. The soluble fraction was transferred to ultracentrifuge tubes and centrifuged in a Ti70 rotor at 55000 rpm (222592 x g) under <20 micron vacuum for 1.5 hr at 4 °C. The pellet was resuspended in TBS buffer using a Potter homogenizer at a ratio of 10 mL buffer per 1 g of pellet. Fos-choline-12 was added to the homogenized membrane fraction to a final volume of 2% (w/v), and the mixture was gently mixed on a nutator for 2 hr at 4 °C. Thereafter, the homogenized membrane fraction was centrifuged in a Ti70 rotor at 41000 rpm (123695 x g) under <20 micron vacuum for 30 min at

4 °C. The soluble fraction containing AgrC-I-His$_6$ was collected and subsequently incubated with Ni-NTA resin (4 mL per 1 L of culture) overnight at 4 °C.

The following purification procedures were performed in a 4 °C cold room. The Ni-NTA-protein mixture was transferred into a 25 mL column, and the flowthrough was collected. The resin was washed with wash buffer 1 (20 mM Tris, 500 mM NaCl, 15 mM imidazole, 1 mM TCEP, 0.05 wt% Fos-choline-14, pH 8) and wash buffer 2 (20 mM Tris, 100 mM NaCl, 25 mM imidazole). The completion of each wash was monitored by measuring absorbance at 280 nm (A280) using a Nanodrop. Each wash was considered complete when the A280 value of the collected eluent matched the background A280 value (A280 = 0.05). Recombinant AgrC-I-His$_6$ was eluted using elution buffer (20 mM Tris, 100 mM NaCl, 300 mM imidazole, 1 mM TCEP, 0.05% Fos choline-14, pH 8). Protein elution and identity were monitored using SDS-PAGE (see **Figure S4A**).

AgrC-I-His$_6$ was concentrated to an A280 value of ~5 using a 30 kDa molecular weight cutoff (MWCO) filter tube (Amicon), followed by further SEC purification using a Superdex 200 10/300 column in TBS buffer. The identity and purity of each fraction were monitored using SDS-PAGE. The fractions containing AgrC-His$_6$ were combined and concentrated until the A280 value of the solution reached ~5. Concentrated protein samples were either used immediately or frozen in liquid nitrogen and stored at -80 °C for future use.

Recombinant expression and purification of membrane scaffold protein. Membrane scaffold protein (MSP) production and purification were performed based on the methods of Ritchie et al. with several modifications.[13] *E. coli* BL21 (DE3) producing membrane scaffold protein MSP1E3D1 (pMSP1E3D1) was streaked onto LB agar plates containing 50 µg/mL kanamycin and incubated at 37 °C overnight. A single colony was picked from the plate and used to inoculate LB medium (30 mL) containing kanamycin (50 µg/mL). The starter culture was incubated at 37 °C with shaking at 200 rpm until the OD_{600} reached 0.4 (approximately 5 hr). TB medium was prepared in 4 L flasks (500 mL/flask) and supplemented with kanamycin (50 µg/mL). Aliquots (7 mL) of starting culture were added to each 4 L flask, and the flasks were incubated at 37 °C with shaking at 200 rpm until the OD_{600} reached 1.2 (approximately 5 hr). Protein expression was induced by addition of IPTG (1 mM final concentration), and the culture was incubated at 28 °C with shaking at 300 rpm for 4 hr. Cells were harvested by centrifugation at 8000 x g at 28 °C, and the cell pellet was stored either overnight at 4 °C for immediate use or frozen in liquid nitrogen and stored at -80 °C for future use.

The following protein isolation and purification procedures were performed in a cold room at 4 °C. The cell pellet was resuspended in phosphate buffer (200 mM Na_2HPO_4, 1% Triton X-100, 1x protease inhibitor, pH 7.5) at a ratio of 5 mL buffer to 1 g cell pellet. The *E. coli* cell membranes were disrupted via sonication (35% amplitude, 4 sec on followed by 1 sec off for 12 min), followed by centrifugation at 30,000 x g for 30 min. The cell debris was removed, and the supernatant was mixed with Ni-NTA resin (4 mL per 1 L of culture) and stirred for 1 hr. The mixture was transferred to an empty 25 mL column, and the flowthrough was collected. The resin was washed with 50 mL of wash buffer 1 (40 mM Tris buffer, 300 mM NaCl, 1% Triton X-100, pH 8), followed by 50 mL of wash buffer 2 (40 mM Tris buffer, 300 mM NaCl, 20 mM imidazole, 50 mM sodium cholate, pH 8), and finally 50 mL of wash buffer 3 (40 mM Tris, 300 mM NaCl, 40 mM imidazole, pH 8). The completion of each wash was monitored by measuring A280 using a Nanodrop. Each wash was considered complete when the A280 value of the collected eluent matched the background A280 value (A280 = 0.05). MSP was eluted from the column with 50 mL of elution buffer (40 mM Tris, 300 mM NaCl, 400 mM imidazole, pH 8), and the completion of elution was checked via the Nanodrop. The collected MSP eluent was

transferred into a 10 kDa MWCO dialysis bag and dialyzed twice for 2 hr in 1 L of dialysis buffer (20 mM Tris, 1 mM DTT, pH 8), followed by an overnight dialysis in 2 L of dialysis buffer.

The dialyzed protein was transferred to a 50 mL conical tube. His_7-TEV protease (2 mg) was added to each 50 mL volume of MSP protein (A280 ~ 1.4) to remove the His-tag. The mixture was incubated with gentle rotation on a nutator for 24 hr. The protein mixture was then transferred to Ni-NTA resin (4 mL), and the flowthrough containing MSP was collected. The solution was then concentrated to an A280 value of ~5.0 using a 10 kDa MWCO filter tube (Amicon). The purified MSP was frozen in liquid nitrogen and stored at -80 °C for future usage. MSP isolation and enzymatic His-tag removal steps were monitored with SDS-PAGE (see **Figure S4B**).

AgrC-I nanodisc assembly. AgrC-I nanodiscs were prepared based on the methods of Ritchie et al. and Wang et al. with several modifcations.[12, 13] A 1:3 mixture of POPC:POPG (12.5 mM POPC:37.5 mM POPG) was dissolved in a sodium cholate-HBST buffer (20 mM HEPES, 100 mM NaCl, 1 mM TCEP, 150 mM sodium cholate, pH 7.5) to create a lipid stock solution. A 10% (w/v) Fos-choline-12 solution was also prepared in sodium cholate-HBST. To form empty nanodiscs enclosed by two MSP molecules, MSP + lipids were mixed at a molar ratio of 1:130 MSP:lipid. To assemble AgrC-I dimer nanodiscs, MSP + AgrC-I + lipids were mixed at a molar ratio of 4:1:464 MSP:AgrC-I dimer:lipid to account for lipid displacement by AgrC-I. This ratio was chosen based on prior studies that demonstrated that dimeric AgrC-I is expected to contain 14 transmembrane helices, and each transmembrane helix displaces 4 PO-lipids (56 lipid molecules displaced in total).[12, 13, 16]

Two vials were prepared for AgrC-I dimer nanodisc assembly. The first vial contained MSP (43.6 μM) and lipid mixture (5.67 mM) in sodium cholate-HBST (750 μL). The second vial contained AgrC-I monomer (5.45 μM) and Fos-choline-12 (15 μL of a 10% (w/v) stock for a final concentration of 1% (w/v)) in sodium cholate-HBST (150 μL). The two mixtures were incubated separately on a nutator for 15 min at room temperature. Next, the two mixtures were combined on a nutator for an additional of 30 min at room temperature. In parallel, Bio-Beads were washed in methanol (3x) and then in water (1x) (1 hr per wash), and air dried at room temperature. Bio-Beads (350 mg) were added to the AgrC-I-containing mixture and incubated on a nutator for 1 hr at room temperature. The Bio-Beads were removed via filtration.

To isolate AgrC-I dimer nanodiscs, the mixture was added to Ni-NTA resin (2x volume of the reaction mixture) and the flowthrough was collected. The resin was then washed with wash buffer 1 (20 mM HEPES, 100 mM NaCl, 25 mM imidazole, 1 mM TCEP, pH 8). The completion of each wash was monitored by measuring A280 using a Nanodrop. Each wash was considered complete when the A280 value of the collected eluent matched the background A280 value (A280 = 0.05). The fraction containing nanodiscs was eluted from the Ni-NTA resin with elution buffer (20 mM HEPES, 100 mM NaCl, 300 mM imidazole, 1 mM TCEP, pH 8), and elution completion was monitored using a Nanodrop (as described above). The nanodiscs were concentrated by centrifugation using a 30 kDa MWCO filter tube (Amicon) to an A280 value of ~1.5, followed by a SEC repurification using a Superose 6 10/300 column in HBST buffer. To form empty nanodiscs, the identical protocol for the AgrC-I dimer nanodiscs was followed with two modifications. First, purified AgrC-I was replaced with an equivalent volume of sodium cholate-HBST. Second, SEC purification was performed immediately after the removal of Bio-Beads. Fractions were combined, and the concentration of nanodiscs (both AgrC-I-containing and empty nanodiscs) was adjusted to 5 μM using a MWCO filter (Amicon). The nanodiscs were frozen in liquid nitrogen and stored at -80 °C for future use. Nanodisc assembly and purification were monitored using SDS-PAGE (see **Figure S4C**).

AgrC-I nanodisc competitive histidine kinase assays. Kinase assays on AgrC-I (i.e., autophosphorylation) in the presence of peptide/small molecule ligands were performed according to the method of Muir et al.[12] AgrC-I dimer nanodiscs was diluted to 1.5 µM in TBST buffer (20 mM Tris, 100 mM NaCl, 1 mM TCEP, pH 8). An aliquot (10.5 µL) of AgrC-I nanodisc solution was combined with an aliquot (1.5 µL) of 100 mM $MgCl_2$ solution and an aliquot (1.5 µL) of compound mixture. The compound mixture contained both AIP-I (3.5 µM for CP-20 experiments or 20 µM for AIP-III D4A experiments) and various concentrations of competing ligands or vehicle control dissolved in TBST with 25% (v/v) DMSO. Competing ligands were prepared at 10x concentration in a three-fold dilution series. The positive control compound mixture (i.e., yielding active AgrC-I nanodiscs) contained only AIP-I (3.5 µM for CP-20 experiments or 20 µM for AIP-III D4A experiments) in TBST with 25% (v/v) DMSO. The negative control compound mixture (i.e., yielding inactive AgrC-I nanodiscs) contained TBST with 25% (v/v) DMSO. In parallel, radioactive ^{32}P-labeled ATP (200 µM) was mixed with non-radioactive ATP (200 µM) to obtain an activity of 1 mCi/mL. An aliquot (1.5 µL) of ATP mixture was added to the nanodisc solution to yield a reaction mixture that contained ~1 µM nanodisc, 10 mM $MgCl_2$, and 2.5% total DMSO.

Each reaction mixture was vortexed and briefly centrifuged followed by incubation in a 37 °C water bath for 40 min. In parallel, nitrocellulose membranes (1 x 1 cm square segments) were attached to needles on an iron maiden. The reaction mixtures were incubated on ice for 2.5 min and then vortexed and centrifuged again. Aliquots (4.5 µL) of each reaction mixture were added to nitrocellulose membrane segments and dried for 10 min. The membranes were washed 3x with TBST buffer (10 min per wash).

Each nitrocellulose membrane segment was transferred to a scintillation counting vial containing Bio-Safe-II liquid scintillation counting cocktail (10 mL). The mixture was shaken briefly before measuring ^{32}P radioactivity using a liquid scintillation counter (Beckman LS6500). Radioactivity measurements were normalized to the DMSO (i.e., DMSO radioactivity set to 0% in the absence of AIP-I) and AIP-I (i.e., AIP-I radioactivity set to 100%) controls. Dose-response curves, maximum inhibition values, and potency values were generated using four-parameter non-linear regression analysis ([Inhibitor] vs. response) in GraphPad Prism software (version 10.3.0). Three replicates were collected for each experimental condition.

Fluorescence polarization assays. Purified AgrC-I dimer nanodiscs, TAMRA-AIP-III D4A, and competing compound (from a DMSO stock solution) were combined in PCR tubes (Dot Scientific) such that their final concentrations were 540 nM, 375 nM, and 10 µM, respectively. DMSO was used as a vehicle control. All samples contained a final concentration of 1% (v/v) DMSO. HEPES buffered saline (20 mM HEPES, 100 mM NaCl, 4 µM BSA, 2 µM TCEP, pH 8.0) was added to bring the reaction volume to 50 µL. Reaction tubes were incubated at room temperature for 5 min before being plated into black 384-well microtiter plates (Costar). Fluorescence polarization was measured using a Perkin Elmer EnVision plate reader equipped with a 531 nm excitation filter and 595 nm S-pol and P-pol emission filters. Fluorescence polarization measurements were corrected to the background control containing TAMRA-AIP-III D4A (375 nM) in buffer (i.e., FP probe + buffer polarization set to 0%) and normalized to a DMSO-treated vehicle control containing TAMRA-AIP-III D4A (375 nM) and AgrC-I dimer nanodiscs (540 nM) in buffer (i.e., FP probe + AgrC-I nanodiscs + buffer polarization set to 100%). Four replicates were collected for each experimental condition.

Hemolysis assay protocol.

Overnight cultures of _S. aureus_ USA300 LAC were grown in TSB medium (3 mL). These cultures were diluted 1:100 in fresh, antibiotic-free TSB medium to form inoculating cultures. Inoculating culture was added to test tubes (2 mL/tube). To each culture, aliquots (20 µL) of compounds (derived from stock solutions in DMSO) were added to the desired concentration. DMSO (20 µL) was used as vehicle control. Cultures were grown for 24 hr at 37 °C with shaking at 200 rpm. Aliquots (1 mL) from each culture were transferred into a 24-well clear microtiter plate (Corning). A second aliquot (200 µL) of the remaining culture was used to measure OD_{600} in a clear 96-well microtiter plate (Corning) for each test condition. A suspension of 10% rabbit red blood cells (RBCs) in Alsevers solution (Lampire Biological) was washed by centrifuging at 500 x g for 5 min and resuspending the pellet in PBS buffer to remove preservative antibiotics added by the manufacturer. RBCs were then resuspended in PBS at 1x concentration. Aliquots of RBCs (50 µL) were added to each well in the 24-well plates containing cultures and gently shaken by hand to mix. Plates were then incubated without shaking at 37 °C for 5 min. A portion of culture from each well (500 µL) was centrifuged for 5 min at 500 x g. Aliquots (66 µL) of supernatant from each sample were diluted into fresh TSB medium (134 µL) in clear 96-well microtiter plates (Costar). Absorbance at 405 nm was measured using a Biotek Synergy 2 plate reader running Gen 5 software (version 1.05). Absorbance measurements were corrected to media background absorbance (i.e., media absorbance set to 0% hemolysis) and normalized to OD_{600} (measured in the separate 96-well plate). Three biological replicates were collected for each experimental condition.

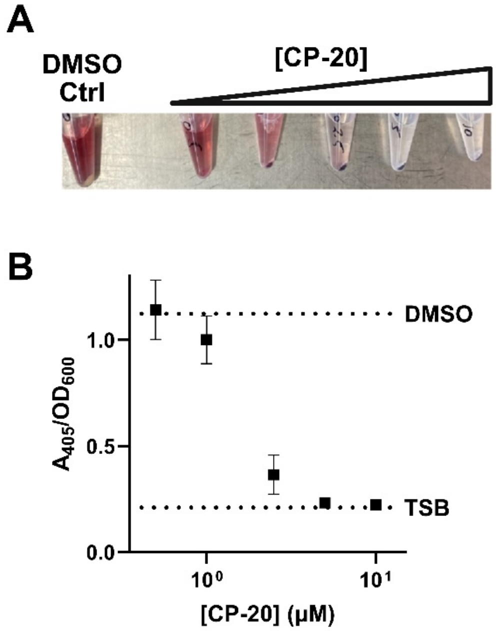

Figure S1. Rabbit red blood cell (RBC) hemolysis assay data. Representative images (A) and quantitation (B) of RBC lysis by *S. aureus* USA300 LAC treated with varying concentrations of CP-20. Dashed lines represent the mean A_{405}/OD_{600} for the DMSO (positive control for hemolysis) and TSB (negative control for hemolysis) controls. Data are shown as mean ± s.e.m. for three biological replicates. See above for details of assay protocol.

Additional text for Figure S1.

We were interested in evaluating CP-20's impact on an *agr*-mediated virulence phenotype. The *agr* system positively regulates the production of pore-forming toxins (most notably α-hemolysin) that enable *S. aureus* to lyse host red blood cells (RBCs).[17] Thus, evaluation of hemolysis is a standard *in vitro* measure of acute *agr*-dependent virulence. We found that CP-20 treatment led to a dose-dependent reduction in hemolysis in the community-associated methicillin-resistant *S. aureus* strain, USA300 LAC (**Figure S1**). This result validates CP-20's bioactivity toward a key *agr*-regulated virulence phenotype.

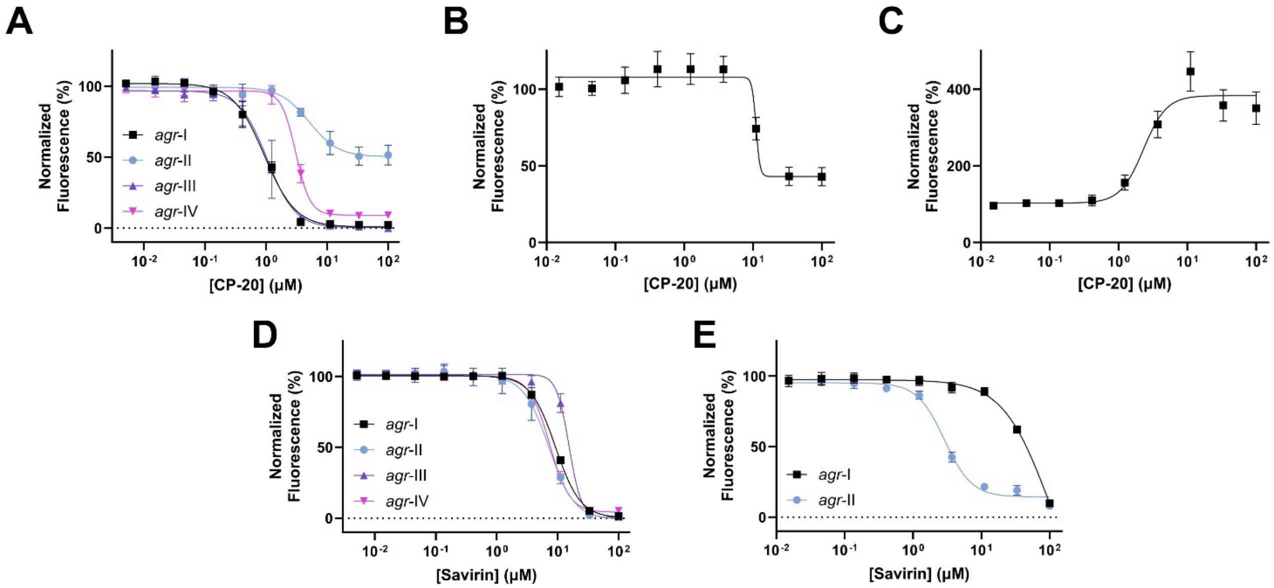

Figure S2. Fluorescent reporter assays for CP-20 and savirin. CP-20 was evaluated in *S. aureus agr* groups I–IV (A), *S. epidermidis agr* group-I (B), and *S. epidermidis agr* group-II (C). Savirin was evaluated in *S. aureus agr* groups I–IV (D) and *S. epidermidis agr* groups I-II (E). The following *S. aureus* reporter strains were used for these experiments: AH1677 (*agr*-I), AH430 (*agr*-II), AH1747 (*agr*-III), and AH1872 (*agr*-IV). The following *S. epidermidis* reporter strains were used for these experiments: AH3408 (*agr*-I) and AH3623 (*agr*-II). All compounds were tested over a range of concentrations in dose-response format. Data are shown as mean ± s.e.m. for three biological replicates. See above for details of reporter assay protocols and strain information.

A

```
Sa-I    MELLNSYNFVLFVLTQMILMFTIPAIISGIKYSKLDYFFIIVISTLSLFLFKMFDSASLIILTSFIIIMYFVKIKWYSILLIMTSQIILYCANYMYIVIYAYITKISDSIFVIFPSFFVV  120
Sa-II   METINNL---AMATFQLVILFTVAKFISFVKFNLRDYFIIVGIIIPTMFLYYFYGSRAVLIPTFSSIIFLFFKLKYYAIVTILVTMIIMYLSNFATVGLFLTLRKYTTDPAILLPLYILS  117
Sa-III  MEALNDYNYVLFVIVQVSLMFFISAFISGIRYKKSDYIYIIGIVLSSVYFFDKIGSISLVVITIFIIIFLYFKIRLYSVFLVMVTQIILYCANFVYIIIFSYIITISHSVFIVLPIFLVV  120
Sa-IV   MESLNSYNFVLFVLTQIILMFTVPSIISGIKYSKSDYLYITGITALSLILFNFIDSVTLIILTIFIIILYFSKIKWYSILLIMTSQIILYCANYMYIVIFTYIVKIVDSIFVIFPIFFVV  120
Se-I    ---MDDINLFPFAGLQIFLMIWVTKVIINMKFNFRDYIIVFTIVIPSAIMYYFWQSKALIVLVIIITIFFYTKIKLYSILVVLFTTMILYITNFITVYIHLTIKDYIPFKFALQLIHFTS  117
Se-II   ---MGKLDFLPFAAIQVFLLVWVTKTIANIKFVGKDYIFITGIIILSAILYNVYASQALVLVVLIIIIFFYSKVRWYSIVIVLMSTLLSYLTNFITVAISLYTENIIHNIYFYNIFHFSI  117

Sa-I    YVTISILFSYIINRVLKKISTPYLILNKGFLIVISTILLLTFSLFFFYSQINSDEAKVIRQYSFIFIGITIFLSILTFVISQFLLKEMKYKRNQEEIETYYEYTLKIEAINNEMRKFRHD  240
Sa-II   FSSVSLLATYLVRISLKKFKKSYLSLNKTYMIIISFVLFATFAFFYIYSTNTSSNGDSLIPYALVFIGLIIFISVVILIMSLFTLKEMKYKRNQEEIETYYEYTLKIEAINNEMRKFRHD  237
Sa-III  YVSISYALAYILNRILKRINGTYLSLNKKFLTVITIVIVITFSLLFAYSQIDASDASTIKQYSLLFLGIIILLSILIFIYSQFTLKEMKYKRNQEEIETYYEYTLKIEAINNEMRKFRHD  240
Sa-IV   YVTISILFSYIINRVLKKISSSYLILNKGFLIVISTILLLTFSLFFFYSQINSDEAKVIRQYSFIFIGITIFLSILTFVISQFLLKEMKYKRNQEEIETYYEYTLKIEAINNEMRKFRHD  240
Se-I    FVIITLIIAYLTQLLFNKLKVSYLSLNKRYLLIITIVLFISFILLYMVSQTDMRGNDTLKLYAILLLGIMVFLSVVILVMSNFTLREMRYKRNVKEIEAYYEYTLRIESINNEMRKFRHD  237
Se-II   FIILSLILAHLFKHLLIRLRYSYLYLSKRYYIIISFVLAIAFIYFYIISQTNLQESNSLNFYAIIFVSITVLLSLVILLLSAFALREMKYKRKLQEIEAYYEYTLRIESINNEMRKFRHD  237

Sa-I    YVNILTTLSEYIREDDMPGLRDYFNKNIVPMKDNLQMNAIKLNGIENLKVREIKGLITAKILRAQEMNFPISIEIPDEVSSINLNMIDLSRSIGIILDNAIEASTEIDDPIIRVAFIESE  360
Sa-II   YVNILTTLSEYIREDDMIGLRAYFNKNIVPMKDNLQMNAIKLNGIENLKVREIKGLITAKILRAQEMNIPISIEIPDEVSSINLNMIDLSRSIGIILDNAIEASTEIDDPIIRVAFIESE  357
Sa-III  YVNILTTLSEYIREDDMTGLRDYFNKNIVPMKDNLQMNALKLNGIENLKVREIKGLLTAKILRAQEMNIPISIEIPDEVTRINLNMIDLSRSIGIILDNAIEASSEIDDPIIRVAFIESE  360
Sa-IV   YVNILTTLSEYIREDDMPGLRDYFNKNIVPMKDNLQMNAIKLNGIENLKVREIKGLITAKILRAQEMSIPISIEIPDEVTHINLNMIDLSRSIGIILDNAIEASTEIDDPIIRVAFIESE  360
Se-I    YVNILTTLSDYIREDDMPGLRKYFNENIVPMKDKLKTRSIKMNGIEKLKVREIKGLITTKIIQAQEKRIPISIEVPDEIDRIDMNTVELSRIIGIIVDNAIEASENLEEPLINIAFIDNE  357
Se-II   YVNILTTLSDYIREDDMPGLRKYFDEHIVPMKDKLKTRSIKMNGIEKLKVREIKGLITTKIIQAQEKRIPISIEVPDEIDRIDMNTVELSRIIGIIVDNAIEASENLEEPLINIAFIDND  357

Sa-I    NSVTFIVMNKCADDIPRIHELFQESFSTKGEGRGLGLSTLKEIADNADNVLLDTIIENGFFIQKVEIINN--  430
Sa-II   NSVTFIVMNKCADDIPRIHELFQESFSTKGEGRGLGLSTLKEIADNADNVLLDTIIENGFFIQKVEIINN--  427
Sa-III  NSVTFIVMNKCADDIPRIHELFQESFSTKGEGRGLGLSTLKEIADNADNVLLDTIIENGFFIQKVEIINN--  430
Sa-IV   NSVTFIVMNKCADDIPRIHELFQESFSTKGEGRGLGLSTLKEIADNADNVLLDTIIENGFFIQKVEIINN--  430
Se-I    ESVTFIVMNKCSNDIPKIHELFEQGFSTKGDNRGLGLSTLKELTDSNENVLLDTVIENGYFVQKVEINNKES  429
Se-II   ESVTFIVMNKCSDDIPKIHELFEQGFSTKGDNRGLGLSTLKELTDSNENVLLDTVIENGYFVQKVEINNKES  429
```

B

	Sa-I	Sa-II	Sa-III	Sa-IV	Se-I	Se-II
Sa-I	100	66.98	76.51	92.79	51.52	51.29
Sa-II	66.98	100	66.28	67.21	56.37	54.48
Sa-III	76.51	66.28	100	77.21	54.8	50.82
Sa-IV	92.79	67.21	77.21	100	53.4	52.93
Se-I	51.52	56.37	54.8	53.4	100	72.73
Se-II	51.29	54.48	50.82	52.93	72.73	100

C

	Sa-I	Sa-II	Sa-III	Sa-IV	Se-I	Se-II
Sa-I	100	30.69	54.15	86.83	28.71	29.21
Sa-II	30.69	100	31.19	32.18	39.6	35.64
Sa-III	54.15	31.19	100	55.12	36.14	28.71
Sa-IV	86.83	32.18	55.12	100	32.18	32.18
Se-I	28.71	39.6	36.14	32.18	100	46.04
Se-II	29.21	35.64	28.71	32.18	46.04	100

Figure S3. AgrC multiple sequence alignment analysis. (A) Multiple sequence alignment for *S. aureus* AgrC-I, -II, -III, and -IV (labeled as Sa-I, Sa-II, Sa-III, and Sa-IV, respectively) and *S. epidermidis* AgrC-I and AgrC-II (labeled as Se-I and Se-II, respectively). (B) Percent identity matrices for full-length AgrC sequences from *S. aureus* and *S. epidermidis*. (C) Percent identity matrices for predicted AgrC transmembrane domain sequences (residues 1-205) from *S. aureus* and *S. epidermidis*. All sequence alignments and identity analyses were conducted using the Clustal Omega Multiple Sequence Alignment Tool.[18]

Table S2. *S. aureus* fluorescent reporter assays for CP-20 halogen derivatives. Compounds were evaluated in *S. aureus* AH1677 (*agr* group-I). See above for details of reporter assay protocol and strain information. CI = 95% confidence interval. n.c. = not calculated, as an upper or lower limit could not be calculated to 95% confidence.

	***S. aureus* (group-I)**	
**Compound**	**IC_{50} [μM] *(95% CI)***	**Max. Inhibition [%]**
CP-20-F	4.2 (*n.c.*)	93
CP-20-Br	1.1 (*0.92-1.2*)	93
CP-20-I	0.18 (*0.17-0.19*)	93

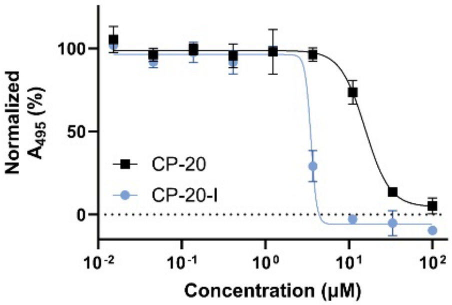

Figure S4. Cell-based AgrC/AgrA activity assays for CP-20 and CP-20-I. β-lactamase reporter (*S. aureus* RN9222) (*P3::blaZ*) assay comparing the activities of CP-20 (data duplicated from Figure 3A in main text) and CP-20-I toward AgrC/AgrA. In this assay, AgrC was activated by exogenously added synthetic AIP-I (100 nM). Data represent the mean ± s.e.m. for three biological replicates. See above for details of reporter assay protocol and strain information.

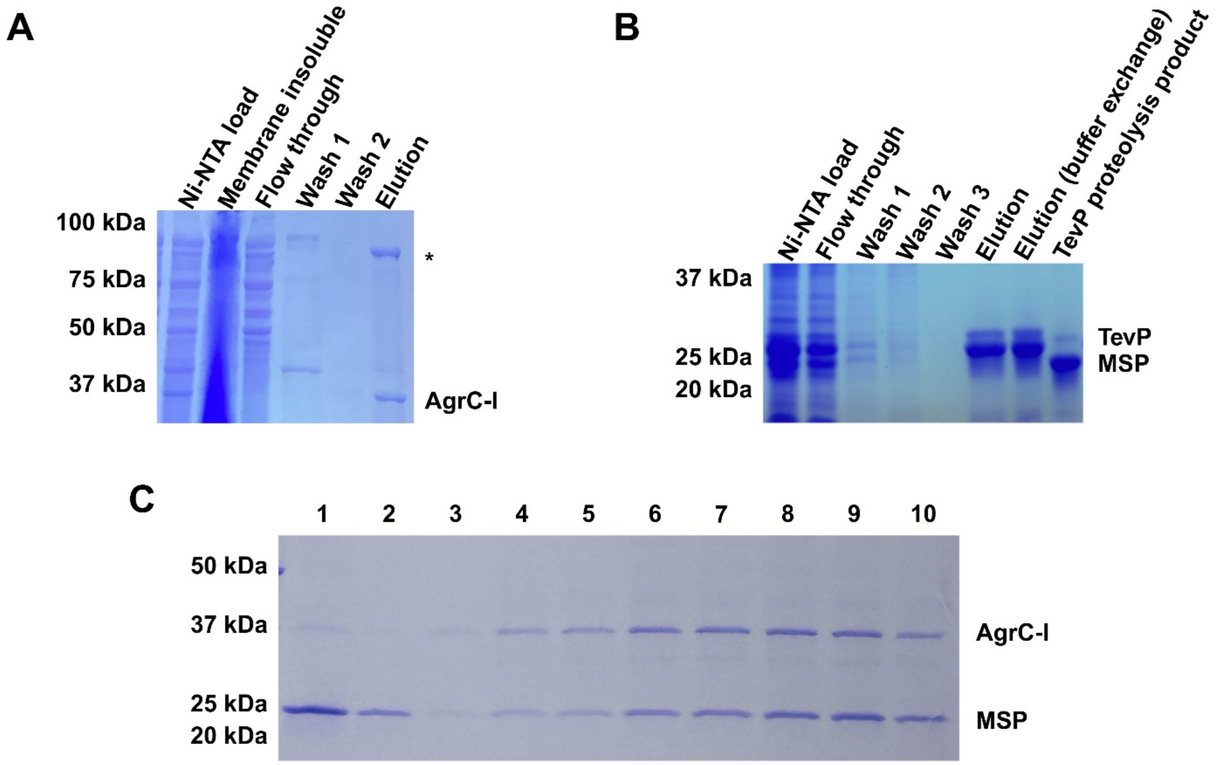

Figure S5. Characterization data for purified recombinant proteins and AgrC-I nanodiscs. SDS-PAGE analysis associated with the purification of recombinant *S. aureus* AgrC-I from *E. coli* membrane extract (A) and membrane scaffold protein (MSP) from *E. coli* soluble lysate (B). Bands corresponding to AgrC-I and MSP are indicated, along with an unknown 100 kDa protein (*) that was co-purified with AgrC-I (reported previously[12]) and TevP, which was co-purified with MSP. Both the 100 kDa protein and TevP were removed upon nanodisc assembly and chromatographic isolation. (C) SDS-PAGE analysis of purified nanodiscs, where lanes 1-10 indicate fractions collected from SEC purification. Bands corresponding to AgrC-I and MSP are indicated. SEC fractions containing both AgrC-I and MSP (fractions 4-10) were combined and concentrated. All gels were visualized with Coomassie brilliant blue.

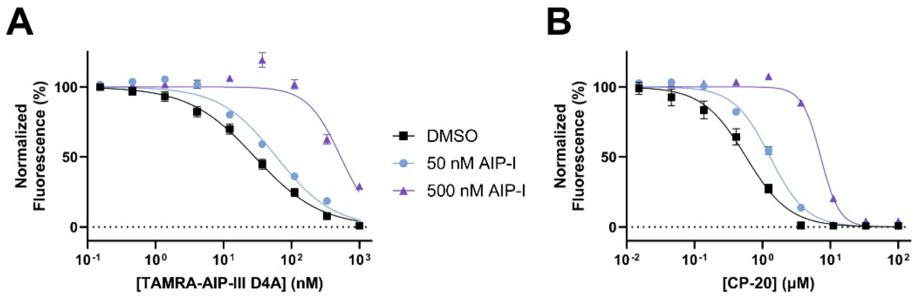

C

Competing Probe ▹	+ 0 nM AIP-I	+ 50 nM AIP-I	+ 500 nM AIP-I
**Compound ▿**		**IC_{50} (nM)**	
TAMRA-AIP-III D4A	28.8	60.3	531
CP-20	574	1300	7220

Figure S6. Cell-based competitive *agr* inhibition assays with TAMRA-AIP-III D4A and CP-20. Fluorescent transcriptional reporter assays with TAMRA-AIP-III D4A (A) and CP-20 (B) performed in the absence of and in competition against synthetic *S. aureus* AIP-I (at 0, 50, or 500 nM). (C) Potency data (given as IC_{50} in nM) for TAMRA-AIP-III D4A and CP-20 in competition with varied concentrations of *S. aureus* AIP-I. *S. aureus* AH1677 (*agr*-I) was used for all experiments. Data represent mean ± s.e.m. for three biological replicates. See above for details of assay protocol and strain information.

Computational methods.

Computational modeling of ligand-AgrC binding. The monomeric structure of *S. aureus* AgrC-I was predicted using AlphaFold2[19] (average pLDDT = 0.7962) and acquired from the Google DeepMind dataset (https://alphafold.ebi.ac.uk/entry/A0A1E8WWT6). Docking poses for *S. aureus* AIP-I, *S. aureus* AIP-III D4A, and CP-20 were modeled using DiffDock,[20] with the predicted AgrC-I structure (as a .pdb file) and SMILES strings for each ligand as input. Docking output files were analyzed and processed in PyMOL.[21]

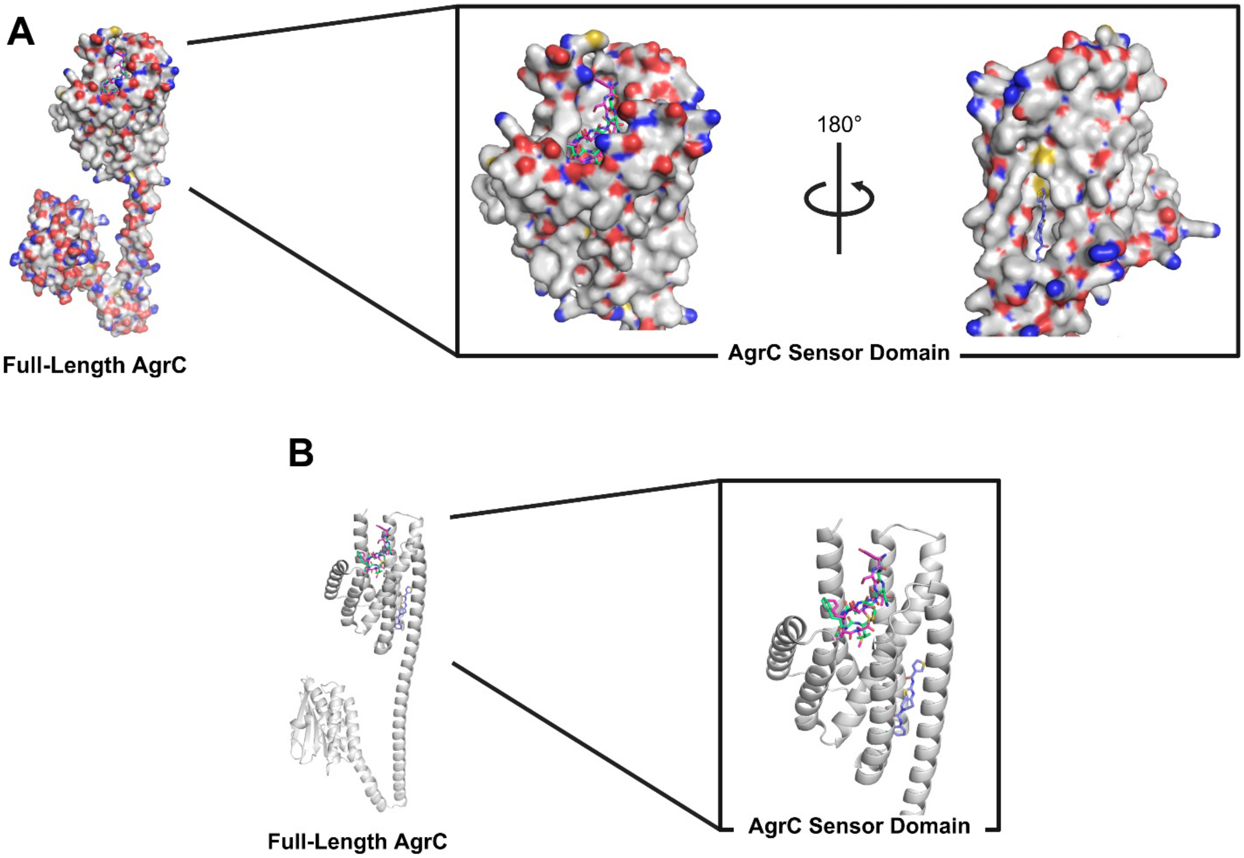

Figure S7. Views of computational models of ligand:AgrC-I binding. Surface (A) and cartoon (B) representations of DiffDock-predicted structures of AIP-I (pink), AIP-III D4A (green), and CP-20 (purple) bound to an AlphaFold2-generated model of *S. aureus* AgrC-I.

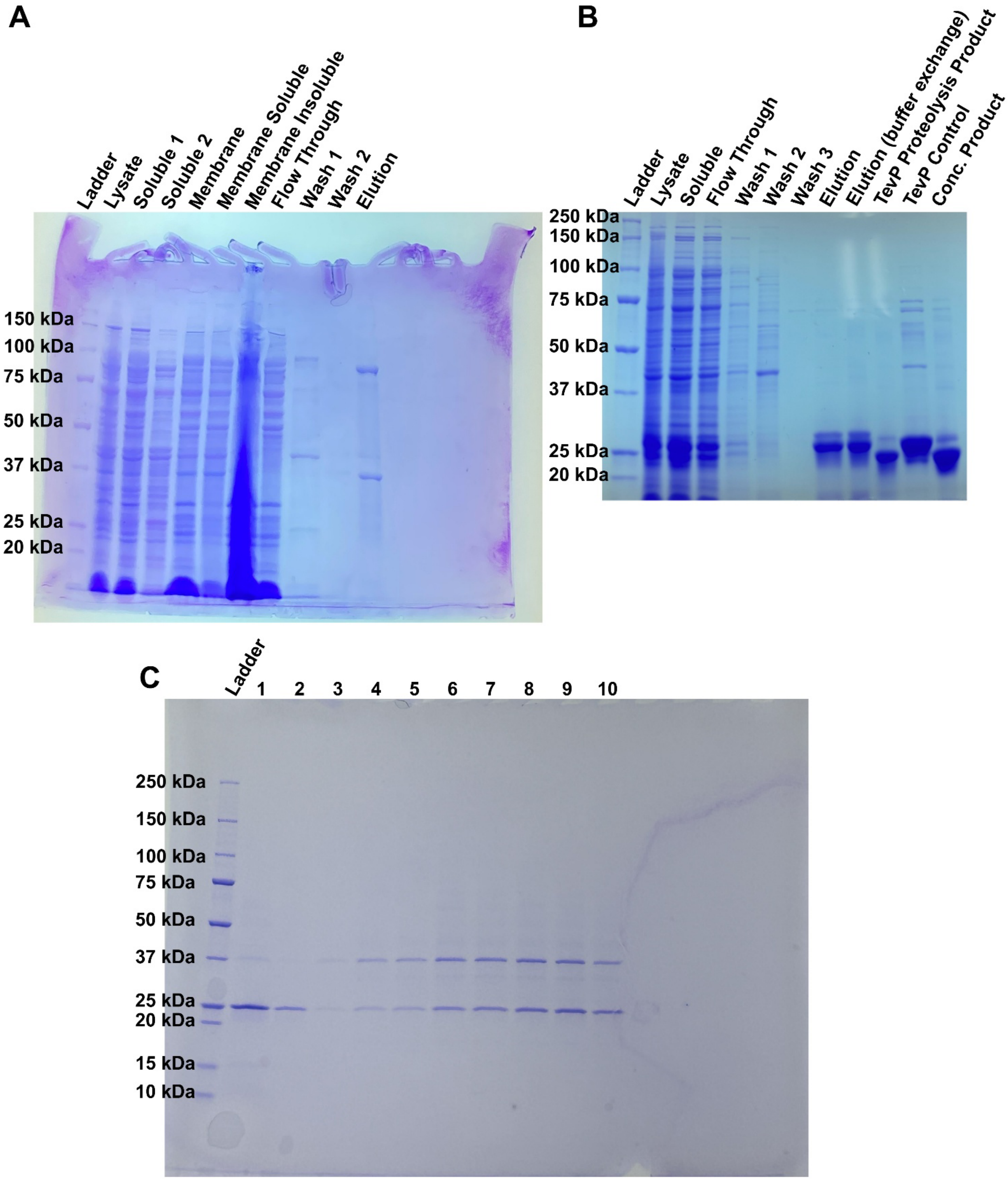

Figure S8. Uncropped gel images from protein and nanodisc purification (for cropped gel images shown **Figure S4**). SDS-PAGE analysis associated with the purification of recombinant (A) *S. aureus* AgrC-I from *E. coli* membrane extract and (B) membrane scaffold protein (MSP) from *E. coli* soluble lysate. (C) SDS-PAGE analysis of purified nanodiscs.

References.

[1] Xiong, X., and Yeung, Y. Y. (2016) Highly *ortho*-Selective Chlorination of Anilines Using a Secondary Ammonium Salt Organocatalyst, *Angew. Chem., Int. Ed. 55*, 16101-16105. https://doi.org/10.1002/anie.201607388

[2] Verho, O., Maetani, M., Melillo, B., Zoller, J., and Schreiber, S. L. (2017) Stereospecific Palladium-Catalyzed C-H Arylation of Pyroglutamic Acid Derivatives at the C3 Position Enabled by 8-Aminoquinoline as a Directing Group, *Org. Lett. 19*, 4424-4427. https://doi.org/10.1021/acs.orglett.7b01776

[3] Tal-Gan, Y., Stacy, D. M., Foegen, M. K., Koenig, D. W., and Blackwell, H. E. (2013) Highly potent inhibitors of quorum sensing in *Staphylococcus aureus* revealed through a systematic synthetic study of the group-III autoinducing peptide, *J. Am. Chem. Soc. 135*, 7869-7882. https://doi.org/10.1021/ja3112115

[4] Eisenbraun, E. L., Vulpis, T. D., Prosser, B. N., Horswill, A. R., and Blackwell, H. E. (2024) Synthetic Peptides Capable of Potent Multigroup Staphylococcal Quorum Sensing Activation and Inhibition in Both Cultures and Biofilm Communities, *J. Am. Chem. Soc. 146*, 15941-15954. https://doi.org/10.1021/jacs.4c02694

[5] Raran-Kurussi, S., Cherry, S., Zhang, D., and Waugh, D. S. (2017) Removal of Affinity Tags with TEV Protease, In *Heterologous Gene Expression in E. coli: Methods and Protocols* (Burgess-Brown, N. A., Ed.), pp 221-230, Springer New York, New York, NY.

[6] Kirchdoerfer, R. N., Garner, A. L., Flack, C. E., Mee, J. M., Horswill, A. R., Janda, K. D., Kaufmann, G. F., and Wilson, I. A. (2011) Structural basis for ligand recognition and discrimination of a quorum-quenching antibody, *J. Biol. Chem. 286*, 17351-17358. https://doi.org/10.1074/jbc.M111.231258

[7] Yarwood, J. M., and Schlievert, P. M. (2003) Quorum sensing in *Staphylococcus* infections, *J. Clin. Invest. 112*, 1620-1625. https://doi.org/10.1172/JCI20442

[8] Malone, C. L., Boles, B. R., and Horswill, A. R. (2007) Biosynthesis of *Staphylococcus aureus* autoinducing peptides by using the synechocystis DnaB mini-intein, *Appl. Environ. Microbiol. 73*, 6036-6044. https://doi.org/10.1128/AEM.00912-07

[9] Lyon, G. J., Mayville, P., Muir, T. W., and Novick, R. P. (2000) Rational design of a global inhibitor of the virulence response in *Staphylococcus aureus*, based in part on localization of the site of inhibition to the receptor-histidine kinase, AgrC, *Proc. Natl. Acad. Sci. U. S. A. 97*, 13330-13335. https://doi.org/10.1073/pnas.97.24.13330

[10] Sully, E. K., Malachowa, N., Elmore, B. O., Alexander, S. M., Femling, J. K., Gray, B. M., DeLeo, F. R., Otto, M., Cheung, A. L., Edwards, B. S., Sklar, L. A., Horswill, A. R., Hall, P. R., and Gresham, H. D. (2014) Selective chemical inhibition of *agr* quorum sensing in *Staphylococcus aureus* promotes host defense with minimal impact on resistance, *PLoS Pathog. 10*, e1004174. https://doi.org/10.1371/journal.ppat.1004174

[11] Olson, M. E., Todd, D. A., Schaeffer, C. R., Paharik, A. E., Van Dyke, M. J., Buttner, H., Dunman, P. M., Rohde, H., Cech, N. B., Fey, P. D., and Horswill, A. R. (2014) *Staphylococcus epidermidis agr* quorum-sensing system: signal identification, cross talk,

and importance in colonization, *J. Bacteriol. 196*, 3482-3493. https://doi.org/10.1128/JB.01882-14

[12] Wang, B., Zhao, A., Novick, R. P., and Muir, T. W. (2014) Activation and inhibition of the receptor histidine kinase AgrC occurs through opposite helical transduction motions, *Mol. Cell 53*, 929-940. https://doi.org/10.1016/j.molcel.2014.02.029

[13] Ritchie, T. K., Grinkova, Y. V., Bayburt, T. H., Denisov, I. G., Zolnerciks, J. K., Atkins, W. M., and Sligar, S. G. (2009) Chapter 11 - Reconstitution of membrane proteins in phospholipid bilayer nanodiscs, *Methods Enzymol. 464*, 211-231. https://doi.org/10.1016/S0076-6879(09)64011-8

[14] Tal-Gan, Y., Ivancic, M., Cornilescu, G., and Blackwell, H. E. (2016) Characterization of structural elements in native autoinducing peptides and non-native analogues that permit the differential modulation of AgrC-type quorum sensing receptors in *Staphylococcus aureus*, *Org. Biomol. Chem. 14*, 113-121. https://doi.org/10.1039/c5ob01735a

[15] Wang, B., Zhao, A., Xie, Q., Olinares, P. D., Chait, B. T., Novick, R. P., and Muir, T. W. (2017) Functional Plasticity of the AgrC Receptor Histidine Kinase Required for Staphylococcal Virulence, *Cell Chem. Biol. 24*, 76-86. https://doi.org/10.1016/j.chembiol.2016.12.008

[16] Wang, L., Quan, C., Xiong, W., Qu, X., Fan, S., and Hu, W. (2014) New insight into transmembrane topology of *Staphylococcus aureus* histidine kinase AgrC, *Biochim. Biophys. Acta 1838*, 988-993. https://doi.org/10.1016/j.bbamem.2013.12.006

[17] Polaske, T. J., West, K. H. J., Zhao, K., Widner, D. L., York, J. T., and Blackwell, H. E. (2023) Chemical and biomolecular insights into the *Staphylococcus aureus agr* quorum sensing system: Current progress and ongoing challenges, *Isr. J. Chem. 63*, e202200096. https://doi.org/10.1002/ijch.202200096

[18] Madeira, F., Madhusoodanan, N., Lee, J., Eusebi, A., Niewielska, A., Tivey, A. R. N., Lopez, R., and Butcher, S. (2024) The EMBL-EBI Job Dispatcher sequence analysis tools framework in 2024, *Nucleic Acids Res. 52*, W521-W525. https://doi.org/10.1093/nar/gkae241

[19] Jumper, J., Evans, R., Pritzel, A., Green, T., Figurnov, M., Ronneberger, O., Tunyasuvunakool, K., Bates, R., Zidek, A., Potapenko, A., Bridgland, A., Meyer, C., Kohl, S. A. A., Ballard, A. J., Cowie, A., Romera-Paredes, B., Nikolov, S., Jain, R., Adler, J., Back, T., Petersen, S., Reiman, D., Clancy, E., Zielinski, M., Steinegger, M., Pacholska, M., Berghammer, T., Bodenstein, S., Silver, D., Vinyals, O., Senior, A. W., Kavukcuoglu, K., Kohli, P., and Hassabis, D. (2021) Highly accurate protein structure prediction with AlphaFold, *Nature 596*, 583-589. https://doi.org/10.1038/s41586-021-03819-2

[20] Corso, G., Stärk, H., Jing, B., Barzilay, R., and Jaakkola, T. (2023) DiffDock: Diffusion Steps, Twists, and Turns for Molecular Docking, In *International Conference on Learning Representations (ICLR)*.

[21] Schrodinger, LLC. (2015) The PyMOL Molecular Graphics System, Version 1.8.
